# Mitochondrial estrogen receptors alter mitochondrial priming and response to endocrine therapy in breast cancer cells

**DOI:** 10.1038/s41420-021-00573-2

**Published:** 2021-07-22

**Authors:** Bahriye Karakas, Yeliz Aka, Asli Giray, Sehime Gulsun Temel, Ufuk Acikbas, Huveyda Basaga, Ozgur Gul, Ozgur Kutuk

**Affiliations:** 1 Sabanci University, Molecular Biology, Genetics and Bioengineering Program, Istanbul, Turkey; 2 Baskent University School of Medicine, Dept. of Immunology, Adana Dr. Turgut Noyan Medical and Research Center, Adana, Turkey; 3 Department of Genetics and Bioengineering, Alanya Alaaddin Keykubat University, Alanya, Turkey; 4 Bursa Uludag University, Faculty of Medicine, Department of Histology and Embryology, Bursa, Turkey; 5 Bursa Uludag University, Faculty of Medicine, Department of Medical Genetics, Bursa, Turkey; 6 Bursa Uludag University, Institute of Health Sciences, Department of Translational Medicine, Bursa, Turkey; 7 Bilgi University, Department of Genetics and Bioengineering, Istanbul, Turkey

**Keywords:** Breast cancer, Apoptosis

## Abstract

Breast cancer is the most common cancer with a high rate of mortality and morbidity among women worldwide. Estrogen receptor status is an important prognostic factor and endocrine therapy is the choice of first-line treatment in ER-positive breast cancer. However, most tumors develop resistance to endocrine therapy. Here we demonstrate that BH3 profiling technology, in particular, dynamic BH3 profiling can predict the response to endocrine therapy agents as well as the development of acquired resistance in breast cancer cells independent of estrogen receptor status. Immunofluorescence analysis and subcellular fractionation experiments revealed distinct ER-α and ER-β subcellular localization patterns in breast cancer cells, including mitochondrial localization of both receptor subtypes. shRNA-mediated depletion of ER-β in breast cancer cells led to resistance to endocrine therapy agents and selective reconstitution of ER-β in mitochondria restored sensitivity. Notably, mitochondria-targeted ER-α did not restore sensitivity, even conferred further resistance to endocrine therapy agents. In addition, expressing mitochondria-targeted ER-β in breast cancer cells resulted in decreased mitochondrial respiration alongside increased total ROS and mitochondrial superoxide production. Furthermore, our data demonstrated that mitochondrial ER-β can be successfully targeted by the selective ER-β agonist Erteberel. Thus, our findings provide novel findings on mitochondrial estrogen signaling in breast cancer cells and suggest the implementation of the dynamic BH3 technique as a tool to predict acquired endocrine therapy resistance.

## Introduction

Breast cancer is a major cause of mortality and morbidity among women in developed and developing countries [1, 2]. Conventional chemotherapy, endocrine therapy agents, and targeted therapies can be utilized as treatment options for breast cancer. However, breast tumors often present with de novo resistance to therapies or develop resistance during the course of the treatment. Understanding the molecular background of these resistance mechanisms is crucial for developing new therapy strategies, improving patients’ quality of life, and for the discovery of the new molecules targeting these resistance mechanisms. ER-α (estrogen receptor-α) and ER-β (estrogen receptor-β) belong to the nuclear receptor family and while they share 96% similarity within their DNA binding domains, there is only 53% similarity in their hormone-binding domains that can explain their differential response to various ligands [3–5]. Furthermore, recent evidence has shown that estrogen receptors also localize to the mitochondria in addition to the nucleus, cytoplasm, and cell membrane in order to regulate fundamental cellular responses [6, 7].

Endocrine therapy, targeting estrogen receptors and estrogen signaling is important approach for the breast cancer therapy. Selective estrogen receptor modulators (SERM), such as tamoxifen and raloxifene bind to ERs, antagonize the effect of estrogen on specific target genes along with a partial agonistic effect. Second, selective estrogen receptor downregulators (SERD) such as fulvestrant which binds to ER and make an irreversible conformational change leading degradation of estrogen receptors, causing reduction in cellular ER-α levels and complete inhibition of ER signaling [8]. Third, aromatase inhibitors (AI), such as Anastrozole inhibits estrogen synthesis by blocking the conversion of androgens to estrogens due to the inhibition of the enzyme aromatase [9].

Activation of the mitochondrial apoptotic pathway in response to chemotherapeutic agents is the most important strategy in targeting cancer cells. Dysregulation of the mitochondrial apoptosis could lead resistance in chemotherapy [10]. Mitochondrial outer membrane permeabilization and release of cytochrome *c* into cytosol is an irreversible event in the apoptotic pathway. This step is regulated tightly by BCL-2 family member proteins [11]. Hence, protein–protein binding code of BCL-2 family member proteins is the main mechanism of regulating mitochondrial outer membrane permeabilization. The binding affinity of anti-apoptotic BCL-2 proteins to each BH3 protein is different and this selective binding pattern enables a novel test called BH3 profiling [12, 13]. The principle of the method is to measure mitochondrial outer membrane permeabilization of cells following the BH3 peptide treatment. The resulting pattern shows how close or far the cell is from the apoptotic threshold [13, 14]. This parameter could be used to predict the chemotherapy response of the cancer cells [15].

According to our knowledge, there is not any test or biomarker to predict de novo or acquired endocrine therapy resistance in breast cancer. Furthermore, there is no study that has been conducted on the impact of mitochondrial estrogen receptors on mitochondrial cell death priming by means of BH3 profiling, so far. Knowing the fact that mitochondria are an essential part of the extrinsic and intrinsic pathways of apoptosis, we considered that mitochondrial estrogen receptors might contribute to endocrine therapy resistance and therefore have a potential of being prominent targets in breast cancer for the near future. In this study, we delineated how mitochondrial estrogen receptor status affects mitochondrial priming and endocrine therapy response in breast cancer cells.

## Results

### Breast cancer cells and normal breast epithelial cells possess distinct ER-α and ER-β expression patterns

**Fig. 1:**
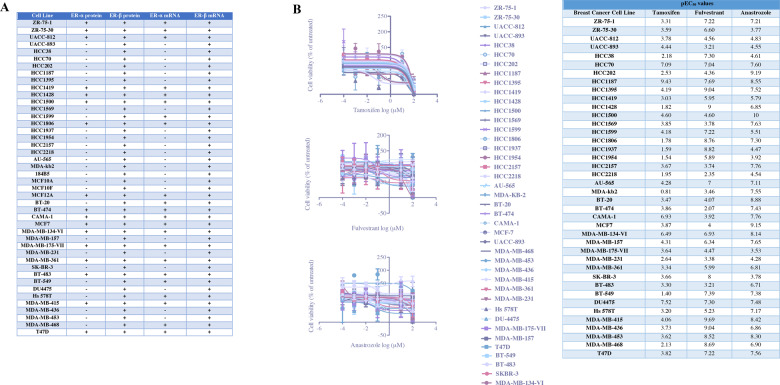
ER-α and ER-β expression profiles and dose-response curves of tamoxifen, fulvestrant, and anastrozole in ATCC breast cancer cell line panel. **A** mRNA and protein expression status of ER-α/-β in breast cancer cells and normal breast epithelial cells were determined by using one-step RT-qPCR and immunoblotting, respectively. **B** Breast cancer cells were exposed to tamoxifen, fulvestrant, and anastrozole for 48 h and cell viability was evaluated by CellTiter-Glo assay (mean ± SEM, *n* = 3). EC_50_ values were determined by nonlinear regression analysis and pEC_50_ values were shown.

First, we verified the ER-α and ER-β status of 44 cell lines in ATCC Breast Cancer Cell Panel out of 45 cell lines. Of note, we could not propagate Hs 578Bst cells in spite of numerous efforts by using different cell culture methods. We screened the cell lines in the breast cancer cell line panel to determine the expression status of ER-α and ER-β both in protein and mRNA levels (Fig. S1A, S1B). All the cell lines screened for ER-β were positive for expression in both protein and mRNA levels showing ubiquitous expression, in contrast to ER-α which is expressed selectively among the cells (Fig. 1A). Our results demonstrated that triple negative (negative for ER-α, PR (progesterone receptor) and HER2 (human epidermal growth factor receptor 2) cell lines [16], including HCC1937, MDA-MB-468, HCC38, HCC70, HCC1187, DU4475, BT-549, Hs578T, MDA-MB-231, MDA-MB-436, MDA-MB-157, MDA-MB-453, BT-20, HCC1395 were actually positive for the expression of ER-β. We found concordance between ER-α protein and mRNA levels except MDA-MB-468, BT-549, Hs 578T, and 184B5 cells having ER-α expression in mRNA level but not in protein level.

Next, we evaluated the response of breast cancer cells and normal breast epithelial cells following exposure to different endocrine therapeutics with distinct mechanisms of action. We performed CellTiter-Glo luminescent cell viability assay at 48 h post-treatment with the indicated concentration of tamoxifen, fulvestrant, or anastrozole to determine the pEC_50_ values. Figure 1B shows dose-response curves of breast cancer cells versus tamoxifen, fulvestrant, and anastrozole, respectively at 48 h post-treatment, in which the % treated cell viability values were compared to those of untreated cells and each time-point was graphed against drug concentration. According to our results, breast cancer cells and normal epithelial cells showed varying pEC_50_ values for tamoxifen, anastrozole, and fulvestrant regardless of their ER-α status and molecular subtypes (Fig. 1C, Fig. S2A). Given that CellTiter-Glo assay could not differentiate cytotoxic and cytostatic effects of endocrine therapeutics, we sought to evaluate the antiproliferative and proapoptotic response of breast cancer cells following drug exposure by additional experimental approaches. As shown in Fig. S3A, treatment of MCF-7, HCC1500, ZR-75-1, CAMA-1, UACC-893, HCC1954, BT-20, and MDA-MB-436 cells with EC_50_ concentrations of tamoxifen, anastrozole, and fulvestrant for each cell line led to decreased proliferation of cells. Correspondingly, treatment with tamoxifen, anastrozole, and fulvestrant resulted in the accumulation of cells in the G1 phase of the cell cycle (Fig. S3B). We next investigated the apoptotic response of cells upon treatment with endocrine therapy agents. As shown in Fig. S3C, exposing MCF-7, HCC1500, ZR-75-1, CAMA-1, UACC-893, HCC1954, BT-20, and MDA-MB-436 cells to tamoxifen, anastrozole or fulvestrant did not activate apoptotic cell death although we observed significant apoptosis induction following treatment with staurosporine. Altogether, these findings demonstrate that endocrine therapy agents act mainly activating cytostatic response in breast cancer cells when used at EC_50_ concentrations for each cell line.

### BH3 Profiling of breast cancer cell lines and normal breast epithelial cell lines reveals distinct patterns of dependence on anti-apoptotic Bcl-2 proteins

**Fig. 2:**
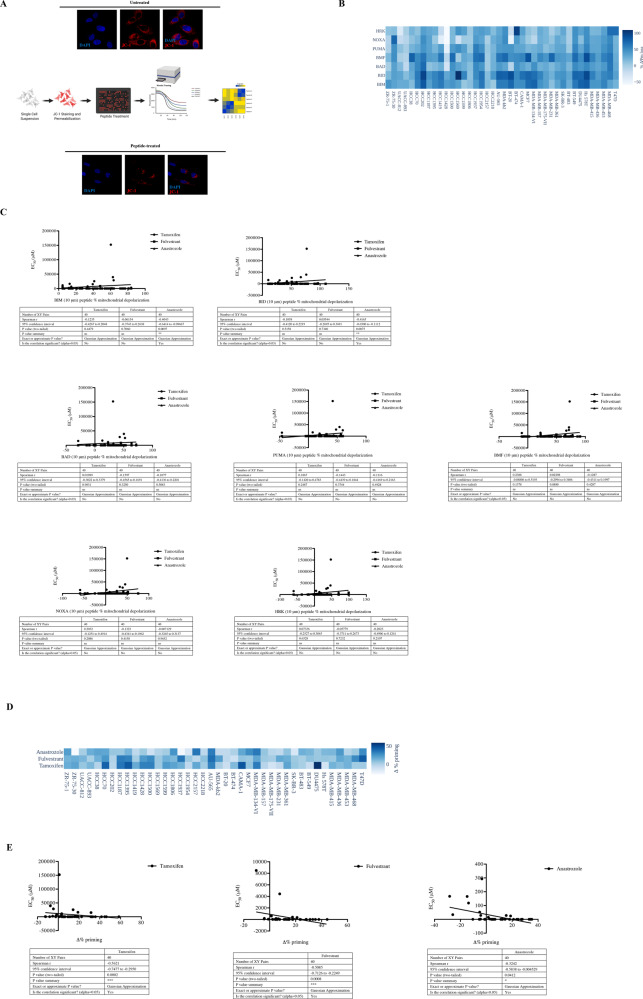
dBH3 profiling assay efficiently predicts response to endocrine therapies in breast cancer cells. **A** Graphical synopsis of BH3 profiling workflow. BH3 profiling measures how close cells are to their apoptotic threshold. Cells are dissociated into single-cell suspensions and stained with JC-1 dye mix and transferred to 384-well black polystyrene plates containing BH3 peptides in MEB assay buffer. JC-1 red fluorescence (Ex: 545 ± 10 nm, Em: 590 ± 10 nm) is monitored for 180 min at 28–32 °C to obtain a kinetic trace. A confocal microscopy image sample is shown, which indicates the loss of JC-1 red fluorescence upon treatment with BIM BH3 peptide. For dBH3 profiling, cells were pretreated with indicated drugs for 16 h and exposed to 1 μM BIM peptide T-EB assay buffer in 384-well black polystyrene plates. After obtaining the kinetic traces for 180 min, area under curve is used to calculate the final BH3 profiles and primed mitochondria are characterized by increased response to BH3 peptides. Data were depicted as heat-map graphs. Illustration was created with BioRender.com. **B** Heat-map of BH3 profiles of breast cancer cells (peptide concentration: 10 μM, *n* = 3). **C** Correlation of BH3 profiles (BID, BIM, BAD, PUMA, BMF, NOXA, and HRK) of 40 breast cancer cell lines with EC_50_ values of tamoxifen, anastrozole and fulvestrant were determined by non-parametric Spearman r correlation test with a two-sided t-test for significance. **D** Heat-map of dBH3 profiles of breast cancer cells following treatment with EC_50_ values of tamoxifen, anastrozole, and fulvestrant for 16 h. **E** Correlation of dBH3 profiles of 40 breast cancer cell lines with EC_50_ values of tamoxifen, anastrozole and fulvestrant was determined by non-parametric Spearman *r* correlation test (*P < 0.05, **P < 0.01 by two-tailed *t* test).

BH3 and dynamic BH3 profiling assays functionally evaluate mitochondrial priming, which indicates the closeness to the threshold at which cells commit to cell death. Both assays have been previously utilized successfully to predict response to conventional chemotherapy and targeted therapies [15, 17–19]. The basic experimental steps of BH3 profiling assays are shown in Fig. 2A. We performed BH3 profiling in breast cancer cell lines and normal breast epithelial cells to detect any endogenous differences in their apoptotic priming by using two different concentrations of BH3 peptides (10 and 100 µM). The utilization of using two different concentrations was based on the fact that each concentration could provide different dynamic ranges of mitochondrial membrane potential loss in response to BH3 peptides. We identified that BH3 profiling with 100 µM peptide concentrations provided a limited dynamic range for mitochondrial cell death priming in breast cancer cells (Fig. S4A). Moreover, we also sought to determine whether apoptotic priming was associated with response to endocrine agents in breast cancer cells. As shown in Fig. S4B, we could not detect any association between the response of BH3 peptides (100 µM) and EC_50_ values of 40 breast cancer cells for endocrine therapy agents. In fact, BH3 profiling with 10 µM peptide concentrations provided an improved dynamic range for differentiating the priming status of cancer cells (Fig. 2B). A significant association was observed with BIM and BID response and anastrozole EC_50_ values of breast cancer cells. Of note, we also found that breast cancer cell lines were more primed than the normal breast epithelial cell lines (Fig. S2B). In order to get further insight into the potential correlation of mitochondrial priming and endocrine therapy responsiveness, we analyzed the dynamic BH3 (dBH3) profiles of breast cancer cells by using EC_50_ concentrations of tamoxifen, anastrozole, and fulvestrant. As demonstrated in Fig. 2D, dBH3 profiling provided different response patterns for each cell line. Consistent with the diverse response of breast cancer cells in dBH3 profiling assay, we detected a significant association of Δ% priming and response to tamoxifen, fulvestrant, and anastrozole (Fig. 2E). In line with BH3 profiling results, breast cancer cell lines were more primed in dBH3 profiling assays when compared to the normal breast epithelial cell lines (Fig. S2C). Collectively, these results demonstrate the potential competency of BH3 profiling techniques to predict response to endocrine agents in breast cancer cells.

### BH3 profiling and dynamic BH3 profiling predict acquired resistance to endocrine therapy reagents

**Fig. 3:**
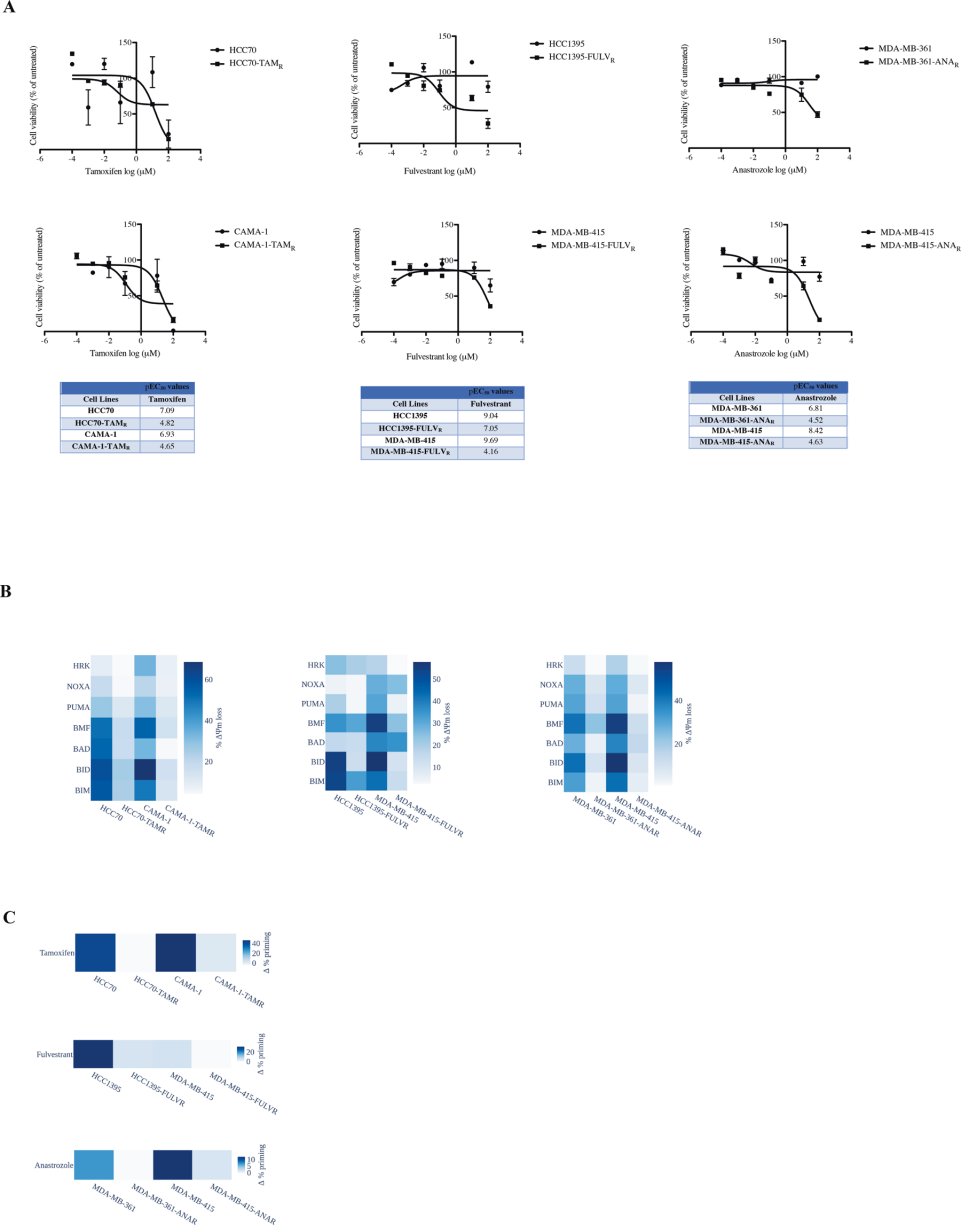
BH3 profiling and dBH3 profiling assays predict the development of acquired resistance to endocrine therapy agents. **A** HCC70, HCC70-TAM_R_, CAMA-1, CAMA-1-TAMR, HCC1395, HCC1395-FULV_R_, MDA-MB-415, MDA-MB-415-FULV_R_, MDA-MB-361, MDA-MB-361-ANA_R_, MDA-MB-415, and MDA-MB-415-ANA_R_ cells were treated with indicated concentrations of tamoxifen, fulvestrant, and anastrozole for 48 h, and cell viability was evaluated by CellTiter-Glo assay (mean ± SEM, *n* = 3). EC_50_ values of parental and resistant cells were determined by nonlinear regression analysis and pEC_50_ values were shown in the tables. Heat-map of (**C**) BH3 profiles (peptides with 10 µM concentrations) and (**D**) dBH3 profiles (BIM with 10 µM concentration) of parental and resistant breast cancer cells.

Because BH3 profiling and dBH3 profiling functionally predict response to endocrine therapy agents, we next evaluated their potential for foreseeing acquired resistance to endocrine therapy. To test this question, we developed tamoxifen-, fulvestrant- and anastrozole-resistant cell lines as previously described [20]. Our results revealed significantly increased pEC_50_ values for tamoxifen, fulvestrant, and anastrozole in resistant cells compared to parental cells (Fig. 3A). As shown in Fig. S5A–C, tamoxifen-, fulvestrant- and anastrozole-induced inhibition of cell proliferation and accumulation at G1 phase in parental cells were abrogated in resistant cells without any effect on apoptotic response. In agreement with these observations, BH3 profiling assays demonstrated that tamoxifen-resistant (HCC70 TAM_R_, CAMA-1 TAM_R_), fulvestrant-resistant (HCC1395-FULV_R_, MDA-MB-415-FULV_R_), and anastrozole-resistant (MDA-MB-361-ANA_R_, MDA-MB-415-ANA_R_) cells were less primed when compared to their parental counterparts (Fig. 3B). In addition, tamoxifen-resistant (HCC70 TAM_R_, CAMA-1 TAM_R_), fulvestrant-resistant (HCC1395-FULV_R_, MDA-MB-415-FULV_R_) and anastrozole-resistant (MDA-MB-361-ANA_R_, MDA-MB-415-ANA_R_) cells also demonstrated decreased dBH3 profiling assay response in comparison to parental cells (Fig. 3C). Taken together, these findings suggest that both BH3 and dBH3 profiling assays can predict the development of acquired resistance to endocrine therapies in breast cancer cells.

### Estrogen receptor isoforms demonstrate distinct mitochondrial localization patterns in breast cancer cell lines and normal breast epithelial cells

**Fig. 4:**
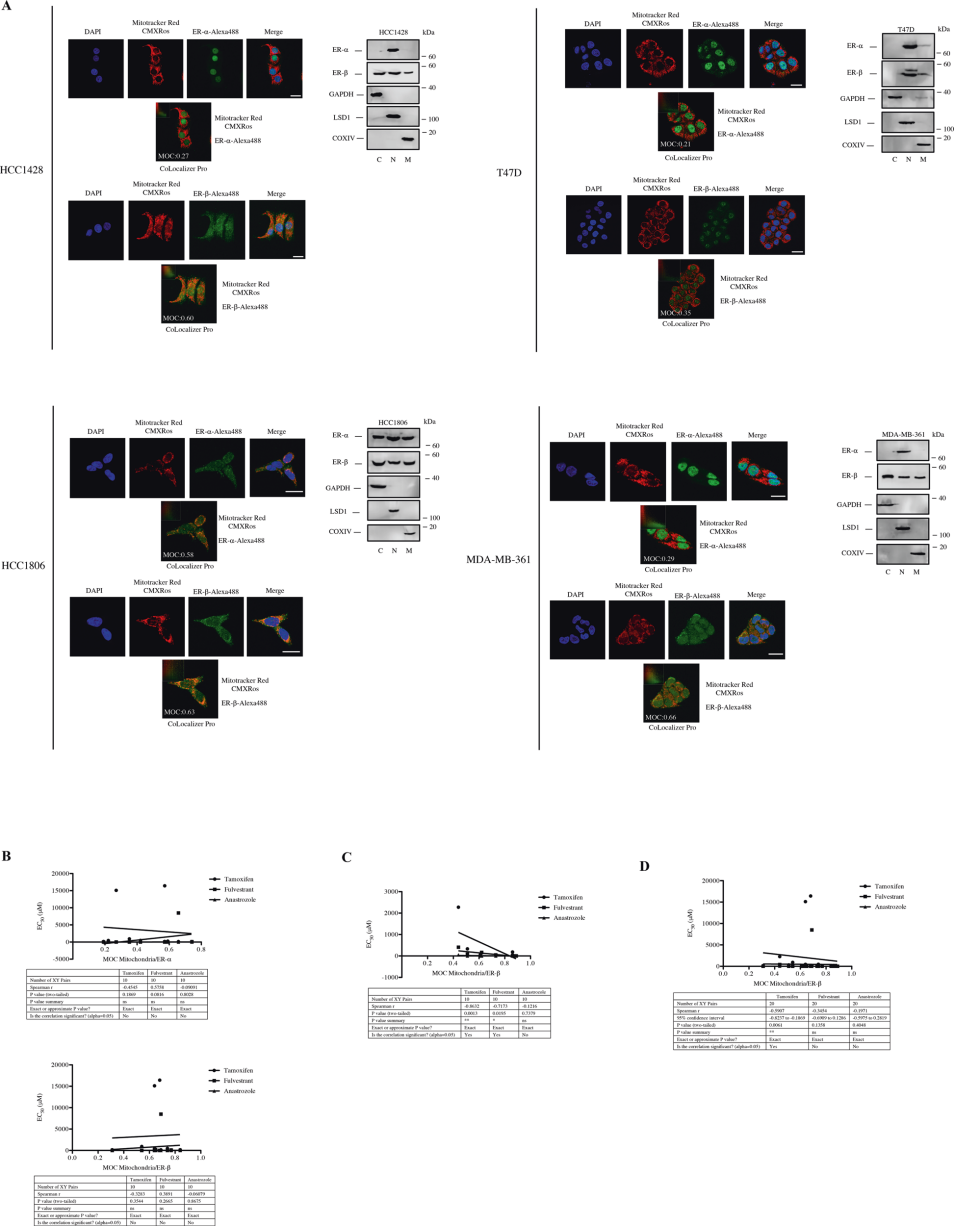
Correlation of mitochondrial localization of ER-α and ER-β with endocrine therapy response in breast cancer cells. **A** Cytoplasmic, nuclear and mitochondrial localization of ER-α and ER-β in HCC1428, T47D, HCC1806, and MDA-MB-361 was evaluated by using immunofluorescence staining and confocal microscopy. DAPI and MitoTracker Red CMXRos were used to visualize the nucleus and mitochondria of the cells, respectively. Scale bars, 10 μm. Colocalization analysis was performed by using CoLocalizer Pro 3.0.2 software to quantify the percent of colocalization of MitoTracker Red CMXRos (pseudocolor red) and Alexa Fluor 488 (pseudocolor green). A sample of colocalization analysis with Manders overlap coefficient (MOC) was shown for each cell line. Scatter plots were shown on the upper left corner of the CoLocalizer Pro analysis images. Cytosolic, nuclear, and mitochondrial fractions were immunoblotted for ER-α and ER-β. GAPDH, LSD1, and COXIV were probed as loading control for cytosolic, nuclear, and mitochondrial fractions, respectively. **B** Correlation of MOC mitochondria/ER-α and MOC mitochondria/ER-β values of 10 ER-α-positive breast cancer cell lines (BT-474, HCC1419, HCC1428, HCC1806, MCF7, MDA-MB-361, T47D, UACC-812, ZR-75-1, and ZR-75-30) with EC_50_ values of tamoxifen, anastrozole and fulvestrant was determined by non-parametric Spearman *r* correlation test. **C** Correlation of MOC mitochondria/ER-β values of 10 ER-α-negative breast cancer cell lines (HCC1599, BT-20, MDA-MB-436, HCC70, AU-565, MDA-MB-157, MDA-MB-231, DU4475, HCC1187, and HCC1569) with EC_50_ values of tamoxifen, anastrozole and fulvestrant was determined by non-parametric Spearman *r* correlation test (*P < 0.05, **P < 0.01 by two-tailed *t* test). **D** Correlation of MOC mitochondria/ER-β values of 20 breast cancer cell lines with different ER-α status (BT-474, HCC1419, HCC1428, HCC1806, MCF7, MDA-MB-361, T47D, UACC-812, ZR-75-1 and ZR-75-30, HCC1599, BT-20, MDA-MB-436, HCC70, AU-565, MDA-MB-157, MDA-MB-231, DU4475, HCC1187, and HCC1569) with EC_50_ values of tamoxifen, anastrozole and fulvestrant were determined by non-parametric Spearman *r* correlation test (*P < 0.05, **P < 0.01 by two-tailed *t* test).

Next, in order to determine mitochondrial localization of ER-α and ER-β in breast cancer cells and normal breast epithelial cells, we performed immunofluorescent staining by using confocal microscopy. As shown in representative staining samples in Fig. 4A and Fig. S6, ER-α was mostly localized to the nucleus in HCC1428, MDA-MB-361, ZR-75-30, and UACC-812 cells, although ER-β staining indicates the presence of ER-β in cytosolic, mitochondrial, and nuclear compartments. We observed mostly nuclear ER-α and ER-β staining in T47D cells, with partial localization in the mitochondrial compartment. In HCC1806, MCF7, and MCF12A cells, ER-α and ER-β were present in cytosolic, nuclear and mitochondrial subcellular compartments. MCF10A cells lack the expression of ER-α and ER-β was localized to cytosolic, nuclear, and mitochondrial compartments. In parallel with confocal staining data, immunoblotting of protein lysates following subcellular fractionation confirmed the distinct subcellular distribution of ER-α and ER-β within breast cancer cells and normal breast epithelial cells (Fig. 4A, S6). To test whether mitochondrial localization of ER-α and ER-β affects response to endocrine therapy response in breast cancer cells, we have chosen 10 ER-α (+)/ER-β (+) and 10 ER-α (−)/ER-β (+) and we analyzed the association of mitochondrial localization of ER-α and ER-β and EC_50_ values of cells for tamoxifen, anastrozole, and fulvestrant. While we did not detect any significant association between mitochondrial ER-α and ER-β and response to endocrine therapies in ER-α (+)/ER-β (+) breast cancer cells (Fig. 4B), we found significant association between sensitivity to tamoxifen and fulvestrant and increased mitochondrial ER-β localization in ER-α (−)/ER-β (+) cells (Fig. 4C). Correspondingly, we identified a significant association between response to tamoxifen treatment and increased mitochondrial ER-β localization when we analyzed the data for 20 cell lines, combined. We also examined whether mitochondrial localization of ER-α and ER-β alters mitochondrial cell death priming. As demonstrated in Figs. S7A and S7B, ER-α and ER-β mitochondrial localization did not correlate with mitochondrial priming in ER-α (+)/ER-β (+) breast cancer cells as determined by BH3 profiling assay. In addition, we could not detect any association between ER-β mitochondrial localization and mitochondrial priming when we analyzed data for 20 cell lines (Fig. S7C). However, we found a positive correlation between ER-β mitochondrial localization and NOXA peptide response in ER-α (−)/ER-β (+) breast cancer cells (Fig. S7D). In keeping with the superior competency of dBH3 profiling for predicting response to endocrine therapy in breast cancer cells, we also investigated whether ER-α and ER-β mitochondrial localization correlate with dBH3 priming status. We found that increased ER-α mitochondrial localization correlates negatively with fulvestrant Δ% priming in ER-α (+)/ER-β (+) breast cancer cells (Fig. S7E), although we did not find any significant association between ER-β mitochondrial localization and Δ% priming (Fig. S7F). When we analyzed the data for all 20 cell lines, we identified correlation between ER-β mitochondrial localization and tamoxifen Δ% priming (Fig. S7G). Furthermore, we found an association between increased ER-β mitochondrial localization and increased tamoxifen and anastrozole Δ% priming in ER-α (−)/ER-β (+) breast cancer cells.

### Mitochondrial ER-α and ER-β differentially modulate response to endocrine therapy and mitochondrial priming

**Fig. 5:**
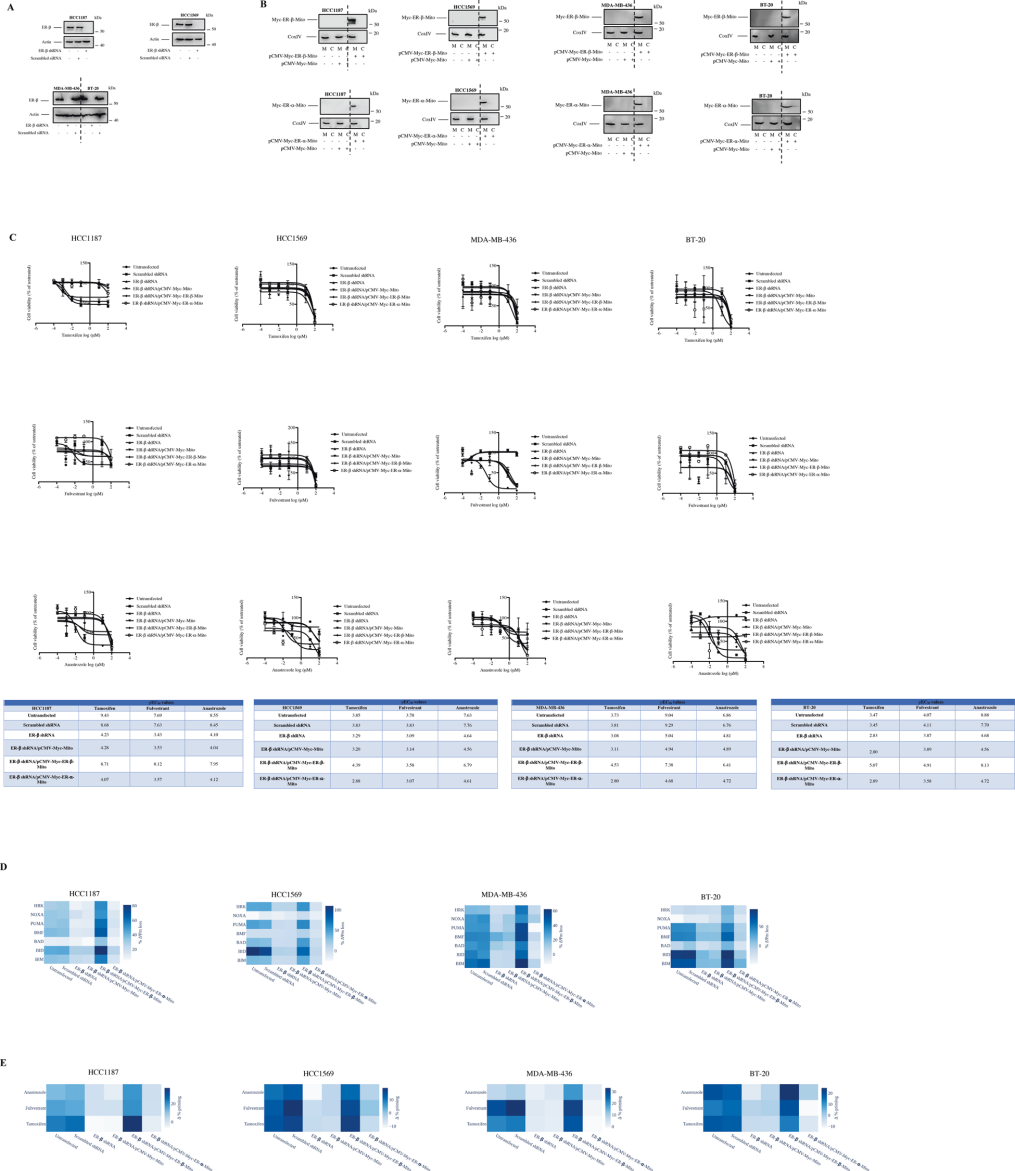
Mitochondrial ER-β prominently alters the response to endocrine therapy in breast cancer cells. **A** ER-α-negative HCC1187, HCC1569, MDA-MB-436, and BT-20 cells were transfected with ER-β shRNA or scrambled shRNA to knockdown ER-β. The efficiency of knockdown was evaluated by using immunoblotting. Actin was probed as loading control. ER-β-silenced HCC1187, HCC1569, MDA-MB-436, and BT-20 cells were transfected with pCMV-Myc-Mito, pCMV-Myc-ER-β-Mito, and pCMV-Myc-ER-α-Mito vectors. Mitochondrial expression of Myc-ER-β-Mito and Myc-ER-α-Mito was confirmed by immunoblotting using Myc-Tag antibody. COXIV was probed as loading control for mitochondrial fractions. **B** Untransfected, scrambled shRNA-transfected, ER-β shRNA-transfected, ER-β shRNA plus pCMV-Myc-Mito-transfected, ER-β shRNA plus pCMV-Myc-ER-β-Mito-transfected, and ER-β shRNA plus pCMV-Myc-ER-α-Mito-transfected cells were treated with tamoxifen, fulvestrant, and anastrozole for 48 h, and cell viability was evaluated by CellTiter-Glo assay (mean ± SEM, *n* = 3). EC_50_ values of cells were determined by nonlinear regression analysis and pEC50 values were shown in the tables. Heat-map graphs of (**C**) BH3 profiles and (**D**) dBH3 profiles of untransfected, scrambled shRNA-transfected, ER-β shRNA-transfected, ER-β shRNA plus pCMV-Myc-Mito-transfected, ER-β shRNA plus pCMV-Myc-ER-β-Mito-transfected and ER-β shRNA plus pCMV-Myc-ER-α-Mito-transfected cells.

To delineate the direct contribution of mitochondrial ER-α and ER-β in endocrine therapy response and mitochondrial priming in breast cancer cells, we depleted ER-β in ER-α (−) HC1187, HCC1569, MDA-MB-436, and BT-20 cells by using shRNA-mediated silencing. As shown in Fig. 5A, ER-β was completely depleted in HC1187, HCC1569, MDA-MB-436, and BT-20 cells transfected with ER-β shRNA. We did not see any difference in ER-β expression in cells transfected with scrambled shRNA. Next, we expressed mitochondria-targeted ER-α and ER-β in ER-β-silenced HC1187, HCC1569, MDA-MB-436, and BT-20 cells. The expression of ER-α-Mito and ER-β-Mito in mitochondrial compartments was evaluated by subcellular fractionation and immunoblotting (Fig. 5B). Of note, mitochondrial expression of ER-α-Mito or ER-β-Mito did not trigger nuclear ERE luciferase activity in HC1187, HCC1569, MDA-MB-436, and BT-20 cells when stimulated with E2, PPT (ER-α agonist) or DPN (ER-β agonist) (Fig. S8). As demonstrated in Fig. 5C, depletion of ER-β led to increased resistance to tamoxifen, fulvestrant, and anastrozole in HC1187, HCC1569, MDA-MB-436, and BT-20 cells. Enforced expression of ER-β-Mito resulted in the restoration of sensitivity to tamoxifen, fulvestrant, and anastrozole, although ER-α-Mito expression led to increased resistance to endocrine therapy agents or did not alter the pEC_50_ values in HC1187, HCC1569, MDA-MB-436, and BT-20 cells. We identified that knockdown of ER-β abrogated the anti-proliferative effects of tamoxifen, fulvestrant, and anastrozole in HC1187, HCC1569, MDA-MB-436, and BT-20 cells (Fig. S9A–D). Furthermore, tamoxifen-, fulvestrant- and anastrozole-induced accumulation of cells at G1 phase of the cell cycle were also abolished in ER-β-depleted cells (Fig. S9E–H). In cells transfected with pCMV-Myc-ER-β-Mito, we found that anti-proliferative effects of tamoxifen, fulvestrant, and anastrozole were restored along with accumulation of cells and G1. By contrast, enforced expression of ER-α-Mito did not significantly alter the cellular response following exposure to tamoxifen, fulvestrant, and anastrozole (Fig. S9A–H). Transfection of cells with scrambled shRNA or empty vector pCMV-Myc-Mito did not exert any significant effect. As shown in Fig. S9I–L, endocrine therapy agents did not activate apoptotic cell death in HC1187, HCC1569, MDA-MB-436, and BT-20 cells when used at EC_50_ concentrations for each cell line. Depletion of ER-β by means of shRNA or transfection of cells with pCMV-Myc-ER-β-Mito and pCMV-Myc-ER-α-Mito did not alter the apoptotic response. Of note, we used staurosporine as a positive control to validate the activity of apoptotic signaling in HC1187, HCC1569, MDA-MB-436, and BT-20 cells. Consistent with the cell viability data, depletion of ER-β in HC1187, HCC1569, MDA-MB-436 and BT-20 cells decreased baseline mitochondrial priming and Δ% priming (Fig. 5D, E). Reconstituting mitochondrial ER-β expression led to restored baseline mitochondrial priming and Δ% priming in HC1187, HCC1569, MDA-MB-436, and BT-20 cells. However, enforced mitochondrial ER-α expression did not exert a similar effect. To assess the specificity of these molecular effects in breast cancer cells, we further expressed mitochondria-targeted ER-α and ER-β tagged with pAcGFP1. We confirmed the expression and mitochondrial localization of pAcGFP1-ER-α-Mito and pAcGFP1-ER-β-Mito in HC1187, HCC1569, MDA-MB-436, and BT-20 cells by digital fluorescence microscopy (Fig. S10A). In line with the data obtained with the Myc-tagged expression vectors, reconstituting mitochondrial ER-β expression by using pAcGFP1-ER-β-Mito vector restored sensitivity to tamoxifen, fulvestrant, and anastrozole (Fig. S10B). Moreover, enforced expression of ER-α by using pAcGFP1-ER-α-Mito did not significantly alter the response to endocrine agents. Complementing these findings, we identified that transfection of cells with pAcGFP1-ER-β-Mito led to enhanced BH3 and dBH3 profiling response in HCC1187, HCC1569, MDA-MB-436 and BT-20 cells (Fig. S10C, D). Collectively, these data suggest that mitochondrial ER-β is a target for endocrine therapy agents in breast cancer cells lacking ER-α expression and mitochondrial ER-β and ER-α differentially regulate response to endocrine therapy and mitochondrial priming.

### Mitochondrial ER-α and ER-β differentially modulate mitochondrial respiration and ROS production in breast cancer cells

**Fig. 6:**
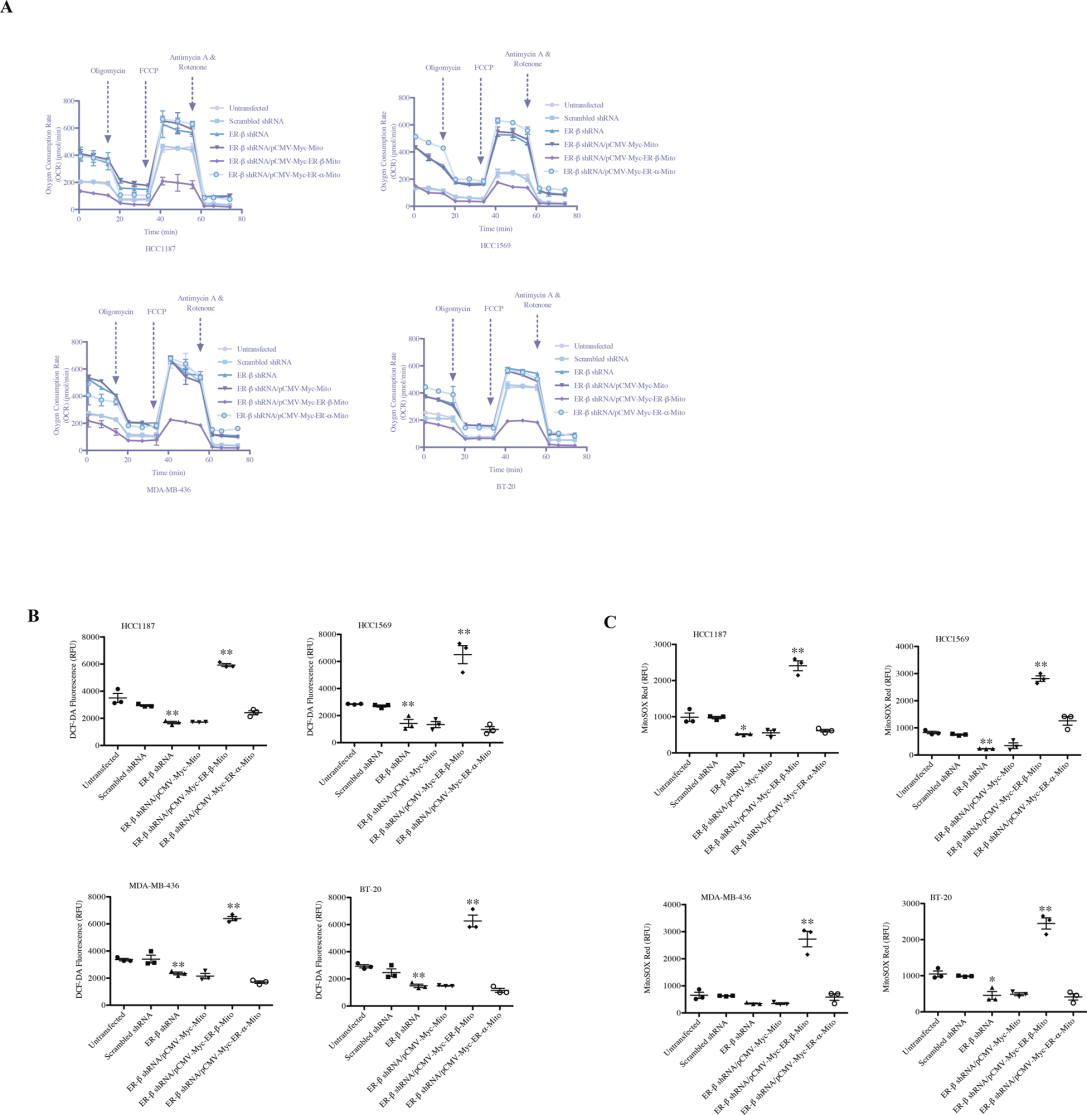
Mitochondrial ER-β and ER-α differentially regulate mitochondrial respiration and ROS production. **A** OCRs (oxygen consumption rates) were determined in untransfected, scrambled shRNA-transfected, ER-β shRNA-transfected, ER-β shRNA plus pCMV-Myc-Mito-transfected, ER-β shRNA plus pCMV-Myc-ER-β-Mito-transfected and ER-β shRNA plus pCMV-Myc-ER-α-Mito-transfected HCC1187, HCC1569, MDA-MB-436, and BT-20 cells. Data were shown as pmol/min. **B** Total ROS production and **C** mitochondrial superoxide production were measured in untransfected, scrambled shRNA-transfected, ER-β shRNA-transfected, ER-β shRNA plus pCMV-Myc-Mito-transfected, ER-β shRNA plus pCMV-Myc-ER-β-Mito-transfected and ER-β shRNA plus pCMV-Myc-ER-α-Mito-transfected HCC1187, HCC1569, MDA-MB-436, and BT-20 cells (mean ± SEM, *n* = 3; *P < 0.05, **P < 0.01 by two-tailed Student’s *t* test, Untransfected vs. ER-β shRNA, ER-β shRNA vs. ER-β shRNA/pCMV-Myc-ER-β-Mito).

Reasoning that expression of mitochondria-targeted estrogen receptors altered sensitivity to endocrine therapy agents and dynamic BH3 profiling response in breast cancer cells, we sought to identify the effect of mitochondrial estrogen receptors on mitochondrial bioenergetics. Thus, we examined mtOXPHOS capacity of ER-β-silenced breast cancer cells transfected with mitochondria-targeted ER-α and ER-β. As seen in Fig. 6A and Fig. S11, shRNA-mediated depletion of ER-β led to increased non-mitochondrial oxygen consumption, basal respiration, and maximum respiration in HC1187, HCC1569, MDA-MB-436, and BT-20 cells. Consistent with these findings, we observed significantly increased ATP-coupled respiration in all four cell lines upon depletion of ER-β by shRNA-mediated knockdown. Furthermore, proton leak was significantly increased only in HCC1569 and BT20 cells and reserve respiratory capacity was unaltered in cells following depletion of ER-β (Fig. 6A and S11). Restoring ER-β expression confined to mitochondria reversed these effects with decreased non-mitochondrial oxygen consumption, basal respiration, and maximum respiration. Our results also showed decreased ATP production. However, we only detected limited effect of enforced mitochondrial ER-α expression on mitochondrial respiration parameters when compared to cells transfected with ER-β shRNA (Fig. 6A and S11). Moreover, depletion of ER-β resulted in decreased total ROS production and mitochondrial superoxide production in HC1187, HCC1569, MDA-MB-436 and BT-20 cells (Figs. 6B and C). As expected, restoring mitochondrial ER-β expression led to increased total ROS and mitochondrial superoxide levels, although mitochondrial ER-α expression did not exert such an effect.

### Selective ER-β agonist Erteberel inhibits cell proliferation and increases dBH3 response by targeting mitochondrial ER-β in breast cancer cells

**Fig. 7:**
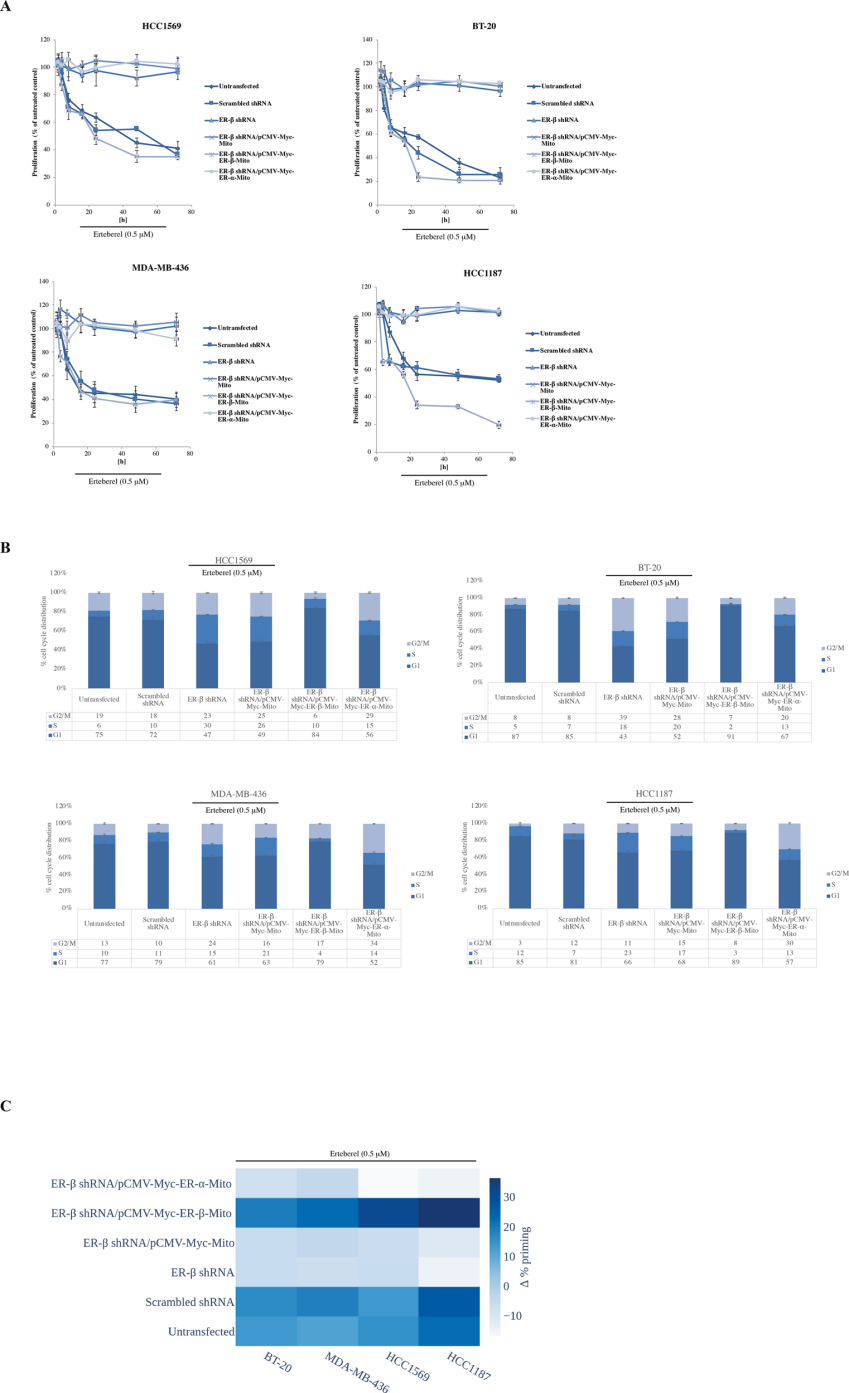
ER-β agonist Erteberel can target mitochondrial ER-β to inhibit cell proliferation and increase mitochondrial priming. **A** Untransfected, scrambled shRNA-transfected, ER-β shRNA-transfected, ER-β shRNA plus pCMV-Myc-Mito-transfected, ER-β shRNA plus pCMV-Myc-ER-β-Mito-transfected and ER-β shRNA plus pCMV-Myc-ER-α-Mito-transfected HCC1187, HCC1569, MDA-MB-436, and BT-20 cells were treated with 0.5 μM Erteberel for 0–72 h. Proliferation of cells was monitored by CyQuant NF proliferation assay (mean ± SEM, *n* = 4). B) Untransfected, scrambled shRNA-transfected, ER-β shRNA-transfected, ER-β shRNA plus pCMV-Myc-Mito-transfected, ER-β shRNA plus pCMV-Myc-ER-β-Mito-transfected and ER-β shRNA plus pCMV-Myc-ER-α-Mito-transfected HCC1187, HCC1569, MDA-MB-436 and BT-20 cells were treated with 0.5 μM Erteberel 16 h. Cell cycle profiles of untreated and treated cells were determined by BD Cycletest Plus DNA assay kit on FACSCanto flow cytometer. Data were shown as % of cells in G2/M, S, and G1 phases representing three independent experiments. **C** Heat-map of dBH3 profiles of untransfected, scrambled shRNA-transfected, ER-β shRNA-transfected, ER-β shRNA plus pCMV-Myc-Mito-transfected, ER-β shRNA plus pCMV-Myc-ER-β-Mito-transfected and ER-β shRNA plus pCMV-Myc-ER-α-Mito-transfected HCC1187, HCC1569, MDA-MB-436 and BT-20 cells following treatment with 0.5 μM Erteberel for 16 h.

Next, we tested whether selective ER-β agonist Erteberel (LY500307) could target mitochondrial ER-β pool to exert its activity against the receptor. Of note, Erteberel was previously shown to be effective as an anti-tumor agent against triple-negative breast cancer, melanoma, and glioblastoma [21, 22]. In Fig. 7A, we showed that Erteberel treatment led to decreased cell proliferation in HC1187, HCC1569, MDA-MB-436, and BT-20 cells, and depletion of ER-β abrogated this effect. We then demonstrated that enforced expression of mitochondria-targeted ER-β resensitized HC1187, HCC1569, MDA-MB-436 and BT-20 cells to Erteberel. By contrast, reconstituting mitochondrial expression of ER-α failed to alter Erteberel response in cells (Fig. 7A). Treatment with 0.5 μM Erteberel resulted in the accumulation of cells at G1 phase of the cell cycle, which was abrogated in cells transfected with ER-β shRNA (Fig. 7B). In line with the cell proliferation data, mitochondrial re-expression of ER-β in HC1187, HCC1569, MDA-MB-436 and BT-20 cells led to restored effect of Erteberel on cell cycle distribution. In contrast, mitochondrial re-expression of ER-α further enhanced the effects of ER-β shRNA with increased accumulation of cells at G2/M (Fig. 7B). Of note, dBH3 profiling experiments following treatment with 0.5 μM Erteberel clearly demonstrated decreased Δ% priming upon depletion of cells with ER-β shRNA, which was restored by enforced expression of mitochondria-targeted ER-β, but not by mitochondria-targeted ER-α (Fig. 7C). Taken together, our results supported the role of mitochondrial ER-β as a target for selective ER-β agonists in breast cancer cells lacking ER-α expression.

## Discussion

ER-α and ER-β have been previously shown to control the transcription of several genes in response to estrogen signaling and deregulation of these signaling pathways can contribute to the development of breast cancer [23]. Recently, ER-β has been identified as a novel target for endocrine therapy and a mediator of estrogen action in breast cancer cells with tumor-initiating capabilities [24]. In some studies, ERβ has been linked with improved survival in endocrine therapy such as tamoxifen and fulvestrant alone was insufficient to block proliferation, the addition of ER-β inhibitor could accomplish the action [24, 25]. We report herein that ER-β is expressed ubiquitously while ER-α is expressed selectively in breast cancer cells and normal breast epithelial cell lines. There is an urgent need for biomarkers that identify accurately which cancer patients will benefit from specific therapies with the goal of personalized medicine. Our present results demonstrated that tamoxifen, fulvestrant, and anastrozole were effective against breast cancer cells regardless of ER-α and ER-β expression status, even if the mechanism of action is different for each endocrine therapy agent. BH3 profiling and dynamic BH3 profiling have been efficiently used to determine anti-apoptotic BCL-2 protein dependency and to predict therapy response in hematologic malignancies and solid tumors [15, 17, 26, 27]. Even though baseline mitochondrial priming by means of BH3 profiling failed to provide an efficacious prediction for endocrine therapy response, dynamic BH3 profiling was more effective. Since these techniques were developed to predict mitochondrial cell death response, this was an expected outcome due to the cytostatic effects of endocrine therapy agents. Notably, both BH3 profiling and dBH3 potently predicted acquired resistance to endocrine therapy agents (Fig. 3B, C).

In addition to their canonical functions as transcription factors, ER-α and ER-β have been shown to localize in mitochondria in breast cancer cells and to regulate several critical cellular processes including cell death and formation of reactive oxygen species and mitochondrial respiration [7, 28–30]. We also found a similar subcellular distribution pattern of ER-α and ER-β in breast cancer cells and normal breast epithelial cells (Fig. 4A and Fig. S6). Importantly, we found that mitochondrial ER-β pool affects response to tamoxifen and fulvestrant in breast cancer cells lacking ER-α. Biochemical data support this notion as we found that mitochondrial ER-α and ER-β differentially affects response to endocrine therapy agents, mitochondrial priming, mitochondrial respiration, and ROS production in breast cancer cells.

The majority of endocrine therapy act by binding to estrogen receptors while some of their effects could be ER-independent [31–33]. Tamoxifen and fulvestrant have been developed as molecules directly binding and repressing to estrogen receptors, although anastrozole acts by inhibiting aromatase. Recently, anastrozole was shown to directly bind to ER-α [34]. Therefore, our findings suggest that mitochondrial estrogen receptors, in particular ER-β, can be directly targeted by endocrine therapy agents. As selective ER-β agonist Erteberel triggered similar effects on mitochondria-targeted ER-β-expressing breast cancer cells, endocrine therapy agents act as agonists on mitochondrial ER-β.

Our work highlights the promising potential of using BH3 profiling assays as unique precision tools to monitor the development of acquired resistance against endocrine therapy agents in breast cancer. Lastly, a better understanding of non-genomic, non-transcriptional estrogen signaling pathway components and discovering of the mechanism of endocrine therapy resistance will provide us the knowledge to develop more effective therapeutic options in breast cancer.

## Materials and methods

### Chemicals

Tamoxifen, fulvestrant, anastrozole, staurosporine, E2 (17β-Estradiol), DPN (2,3-Bis(4-hydroxyphenyl)propionitrile) and PPT (1,3,5-Tris(4-hydroxyphenyl)−4-propyl-1H-pyrazole) were purchased from MilliporeSigma (St Louis, MO, USA). Erteberel (LY500307) was purchased from MedChemExpress (NJ, USA).

### Cell culture

ATCC Breast Cancer Cell Panel (ATCC® 304500 K™) was purchased from the ATCC/LGC Standards (Wessel, Germany). HCC1500, BT-483, HCC-202 cells were grown in RPMI −1640 and UACC 812, UACC-893, MDA-MB-415 cells were grown in Leibovitz (L-15). All the other cell lines were grown in DMEM/F12 medium supplemented with 10% heat-inactivated fetal bovine serum (Sigma, St Louis, MO, USA), 100 IU/ml penicillin, and 100 μg/ml streptomycin (ThermoFisher Scientific, Carlsbad, CA, USA) in a humidified incubator at 37 °C with or without 5% CO_2_. Tamoxifen-resistant HCC70 and CAMA-1, fulvestrant-resistant HCC1395 and MDA-MB-415 and anastrozole-resistant MDA-MB-361 and MDA-MB-415 cells were derived as described before [20]. After selecting resistant clonal lines, cells were maintained in the presence of tamoxifen, fulvestrant or anastrozole plus 5 μg/mL verapamil. Drugs were withdrawn from growth media for 24 h before experiments to reduce the possibility of drug-mediated variations in gene expression. Before each experiment, cells were counted by automatic cell counter and seeded in 96-well, 6-well, 12-well, 60 mm, or 100 mm culture plates.

### Protein isolation and immunoblotting

For total protein extraction, cell lysates were prepared in 1% CHAPS lysis buffer containing protease inhibitors (cOmplete ULTRA, Roche), 5 mM MgCl_2_, 140 mM NaCl, 1 mM EGTA, 1 mM EDTA, 1% CHAPS, 20 mM Tris-HCl [pH = 7.5] for 20 min, followed by centrifugation at 4 °C, 13200 rpm for 10 min. The supernatant was collected as total protein extract and stored at −80 °C for immunoblotting analysis. Protein concentrations were determined by Quick-Start Bradford protein assay (Bio-Rad, Munich, Germany). Proteins (30–100 μg) were mixed with loading buffer and separated on 10% SDS-PAGE and transferred onto PVDF (Millipore) membranes. Membranes were then blocked with 5% non-fat dry milk (AppliChem GmbH, Darmstadt, Germany) in PBS-Tween20, incubated with appropriate primary antibodies overnight, followed by washing in PBS-Tween20 and incubation with HRP-conjugated secondary antibodies (Amersham Pharmacia Biotech, Freiburg, Germany) in antibody buffer containing 10% (v/v) Milk Diluent/Blocking concentrate (KPL). Immunoblots were developed with Luminata Crescendo Western HRP substrate (Millipore) and imaged with C-DiGit Blot Scanner (LI-COR Biosciences, Bad Homburg, Germany) on chemiluminescence mode. Antibodies used for immunoblotting were as follows: ER-α (#8644, Cell Signaling), ER-α (#sc-8002, Santa Cruz), ER-β (#sc-390243, Santa Cruz), β-Actin (#8457, Cell Signaling), GAPDH (#5174, Cell Signaling), CoxIV (#4850, Cell Signaling), LSD1 (#2184, Cell Signaling), BAK (#3814, Cell Signaling), Myc-Tag (#2276, Cell Signaling).

### RNA isolation and real-time one-step RT-PCR analysis

Breast cancer cells were seeded in 60 mm culture plates (10^7^ cells/plate) for RNA isolation. Total cellular RNA from untreated cells was isolated with RNeasy Plus Mini Kit (Qiagen, Hilden, Germany). Qiagen QuantiTect SYBR Green RT-PCR kit and Roche LightCycler 480 were used according to the manufacturers’ instructions. QuantiTect Primer Assays for ER-α (QT00044492, Qiagen), ER-β (QT00060641, Qiagen), and GAPDH (QT00079247, Qiagen) were purchased from Qiagen. PCR conditions were 50 °C for 30 min, 95 °C for 15 min, and 40 cycles of 94 °C for 15 s, 55 °C for 30 s and 72 °C for 30 s. CT values were normalized by subtracting the CT value of the housekeeping gene GAPDH from the CT value of the target genes (ΔCT). The normalized fold change of mRNA expression was expressed as 2^-ΔΔC^_T_, where ΔΔC_T_ = ΔC_T_ sample −ΔC_T_ control [35]. Amplification specificity was confirmed with melt curve analysis and agarose gel electrophoresis of the reaction products.

### Cell viability and cell death assays

CellTiter-Glo Luminescent Cell Viability Assay (Promega) is used to determine the number of viable cells in culture based on quantification of ATP present which reflects the metabolically active cells. For the cell viability assay, the breast epithelial and breast cancer cells were seeded in opaque-walled 96-well cell culture plates (3 ×1 0^5^−5 × 10^5^ cells/well). Tamoxifen, fulvestrant and anastrozole was applied for 48 h in 6-log concentrations (10^−4^, 10^−3^, 10^−2^, 10^−1^, 10, and 100 µM). In addition to untreated control wells containing cells but no drug, control wells containing medium without cells were used to obtain a value for background luminescence. Luminescence was recorded by using Spectramax Gemini XPS (Molecular Devices). The results were obtained from three independent technical repeats and analyzed by non-linear regression analysis. EC_50_ and pEC_50_ values were determined by using GraphPad Prism 5.0 software.

### BH3 profiling and dynamic BH3 profiling

JC-1 (Molecular Probes, USA) plate-based BH3 profiling was done as described before [27, 36]. GeneCust Europe synthesized the peptides used in this assay and peptide sequences were previously described [15, 27]. Briefly, cells were permeabilized and stained in MEB buffer (150 mM Mannitol, 10 mM HEPES-KOH (pH 7.5), 50 mM KCl, 0.02 mM EGTA, 0.02 mM EDTA, 0.1 % BSA, 5 mM Succinate) in the presence of 25 μM digitonin, 5 mM β-mercaptoethanol, 10 μg/ml oligomycin and 1 μM JC-1 for 10 min. Cells were transferred to Corning 384-well Low Flange Black Flat Bottom polystyrene microplates containing BH3 peptides in two different concentrations (100 μM or 10 μM). JC-1 fluorescence (Ex: 545 ± 10 nm, Em: 590 ± 10 nm) was analyzed by using Spectramax Gemini XPS microplate spectrofluorometer in every 5 min for 3 h at 28–32 °C. The area under each curve was calculated and % depolarization was derived by using the following equation: % depolarization = 1-(sample-FCCP)/(DMSO-FCCP). Data shown are mean ± SEM of three independent experiments in duplicate and expressed as % ΔΨm loss compared with DMSO-treated cells. FCCP was used as a positive control. Dynamic BH3 profiling was performed as described before [17]. In brief, 15 μl of BIM peptide (final concentration of 1 μM) in T-EB buffer (300 mM Trehalose, 10 mM HEPES-KOH (pH 7.7), 80 mM KCl, 1 mM EGTA, 1 mM EDTA, 0.1% BSA, 5 mM Succinate) were transferred to 384-well black plates. Following indicated treatment with drugs for 16 h, one volume of the 4× cell suspension in T-EB buffer was added to one volume of 4× dye solution (4 μM JC-1, 40 μg/ml oligomycin, 0.02% digitonin, 20 mM β-mercaptoethanol) for 10 min. Fifteen microliters of 2× cell/dye mix was transferred to each treatment well of the 384-well black plate (final cell number of 2 × 10^4^ cells/well), shaken 15 s and JC-1 fluorescence (Ex: 545 ± 10 nm, Em: 590 ± 10 nm) was analyzed by using Spectramax Gemini XPS microplate spectrofluorometer in every 5 min for 3 h at 28–32 °C. Δ% priming depolarization was derived by using the following equation: Δ% priming = (% priming^treated^−%priming^untreated^).

### shRNA and plasmid transfections

Cells were transfected with ER-β shRNA (ESR2 SureSilencing shRNA Plasmid, 336313KH00992N, Qiagen) a mixture of four plasmids containing ER-β-specific insert sequences (5′-CGCCAGTTATCACATCTGTAT-3′, 5′-TCCCAGCAATGTCACTAACTT-3′, 5′-AGGCCATGATCCTGCTCAATT-3′ and 5′-GATCCTGCTCAATTCCAGTAT-3′) and negative control scrambled shRNA by using Attractene transfection reagent (Qiagen) according to manufacturer’s instructions. Forty-eight hours following transfection, selection of stable transfected cells was performed by using neomycin (G418). Protein knockdown efficiency by shRNA transfection was verified by immunoblotting. pcDNA3-ER-α and pcDNA3-ER-β expression plasmids were kindly provided by Dr. Myles Brown, Dana-Farber Cancer Institute, Boston, USA. pAcGFP1-Mito Vector (GFP expression vector with mitochondria-targeting sequence) was purchased from Clontech. pCMV-Myc-mito was a gift from David Sidransky (Addgene plasmid #71542; http://n2t.net/addgene:71542; RRID: Addgene_71542) [37]. ER-α and ER-β were cloned into pAcGFP-mito and pCMV-Myc-mito expression vectors for expressing ER-α and ER-β in mitochondria. Cells were transiently transfected with indicated GFP-tagged or Myc-tagged ER-α and ER-β vectors by using X-tremeGENE 9 DNA transfection reagent (Roche Applied Science, Indianapolis, IN, USA) according to manufacturer’s instructions. The expression of pCMV-Myc-ER-β-Mito and pCMV-Myc-ER-α-Mito in transfected cells was evaluated by immunoblotting using Myc-Tag antibody. Cells transfected with pAcGFP1-Mito, pACGFP1-ER-β-Mito and pAcGFP1-ER-α-Mito were visualized by using EVOS FLoid digital microscopy system to evaluate the efficiency of transfection.

### Immunostaining and confocal laser scanning microscopy

Cells grown on sterile coverslips (Jena Bioscience circular cover slide (22 mm), # CSL-104) were incubated in 12-well plate format at 37 °C with 100 nM MitoTracker Red CMXRos (ThermoFisher Scientific, #M7512) for 15 min, washed for three times at 5 min per rinse with PBS, fixed for 10 min with 4% (v/v) PFA in PBS, followed by washing with PBS for 5 min. Cell membranes were permeabilized by incubation in 0.05% Triton100-X in PBS for 5 min followed by rinsing with PBS. The fixed and permeabilized cells were incubated with 0.1% BSA in PBS (blocking buffer) for 30 min at room temperature. Coverslips were subsequently exposed to primary antibody 1:100, either ER-α (Santa Cruz, anti-mouse monoclonal antibody, #sc-8002) or ER-β (Santa Cruz, anti-mouse monoclonal antibody, #sc-390243) in blocking buffer for 1 h at room temperature. After washing steps with PBS, coverslips were incubated in dark, with Alexa-488 fluorescence tagged secondary antibody (Alexa Fluor 488 anti-mouse IgG, Invitrogen, Cat #A11034) in blocking buffer for 1 h at room temperature and subsequently rinsed in washing buffer. Cells were incubated without primary antibody (secondary antibody only) to be used as a negative staining control. Cells were mounted with ProLong Antifade mounting medium (Molecular Probes, Eugene, OR, USA) on glass slides (0.17 mm, ThermoFisher Scientific) and visualized by Zeiss LSM 710 confocal microscope through an oil-immersion ×63 objective (excited with 488 nm (for Alexa 488-labeled antibody) and 560 nm (for MitoTracker Red CMXRos-stained mitochondria); light emissions were collected at 530 nm (immunofluorescence for ER-α or ER-β signal), 630 nm (MitoTracker fluorescence) and DAPI (Ex: 405 nm, Em: 459 nm) simultaneously. Control slides were imaged under similar confocal settings. Images were analyzed by using ZEN Lite software (Zeiss) and images converted into.tiff format and transferred to MacOs platform. Colocalizer Pro 3.0.2 software was used for the quantitative analysis (http://www.colocalizer.com/pro.html). For the degree of colocalization (R), we used Manders’ Overlap Coefficient (MOC) [38, 39].

### Dual-luciferase ERE reporter assay

ERE-dependent reporter activity was assayed as described before [40]. Briefly, 2 × 10^4^ cells per well in triplicates in a 96-well plate were transfected with 60 ng Firefly luciferase reporter vector 3X-ERE-TATA-luc and 5 ng control Renilla luciferase vector (pGL4.70 [hRluc], Promega) by using X-tremeGENE 9 DNA transfection reagent (Roche Applied Science, Indianapolis, IN, USA). Firefly and Renilla luciferase levels were quantified using the Dual-Glo Luciferase reporter assay kit (Promega) and Spectramax Gemini XPS microplate spectrofluorometer. Data were shown as relative luminescence units (RLU). 3XERE-TATA-luc was a gift from Donald McDonnell (Addgene plasmid #11354; http://n2t.net/addgene:11354; RRID:Addgene_11354) [40].

### Seahorse XFp cell mito stress test

The real-time measurement of OCR (Oxygen Consumption Rate) and mitochondrial function was performed by using Agilent Seahorse XFp Cell Mito Stress Test kit on the Seahorse XFp Extracellular Flux analyzer (Seahorse Bioscience, Agilent Technologies, Santa Clara, CA, USA) as previously described [41]. 1 μM Oligomycin, 0.5 μM FCCP and 0.5 μM Rotenone/antimycin A injections were used after baseline measurements. Data were shown as pmol/min and analyzed by using Seahorse XF Cell Mito Stress Test Report Generator. The following equations were used to calculate individual test parameters: Non-mitochondrial Oxygen Consumption: Minimum rate measurement after Rotenone/antimycin A injection; Basal Respiration: (Last rate measurement before the first injection)-(Non-Mitochondrial Respiration); Maximal Respiration: (Maximum rate measurement after FCCP injection)- (Non-Mitochondrial Respiration); Proton leak: (Minimum rate measurement after Oligomycin injection)-(Non-Mitochondrial Respiration); ATP Production: (Last rate measurement before Oligomycin injection)-(Minimum rate measurement after Oligomycin injection); Spare Respiratory Capacity (Maximal Respiration)-(Basal Respiration).

### Proliferation and cell cycle assays

Cells (0.5 × 10^3^ cells/well) were plated in black flat-bottom 96-well tissue culture plates. Cellular proliferation was evaluated for 0–72 h by using CyQuant NF Proliferation Assay kit (ThermoFisher Scientific) according to the manufacturer’s instructions by using Spectramax Gemini XPS microplate spectrofluorometer. Results were normalized to untreated cells and shown as % proliferation. Cell cycle analysis was performed by using CycleTEST plus DNA reagent kit (BD Biosciences) according to the manufacturer’s instructions. Cells were analyzed using FACSCanto flow cytometer (BD Biosciences). The percentage of cells in G1, S, and G2/M phases was determined by using the cell cycle analysis module in FlowJo v9 software.

### Subcellular fractionation

Subcellular fractionation was performed as described before [42]. GAPDH was used as cytosolic marker, COXIV was used as mitochondrial marker and LSD1 was used as nuclear marker for immunoblotting experiments.

### Total ROS and superoxide measurements

CM-H_2_DCFDA (ex/em 490/520) and MitoSOX Red (ex/em 510/580) reagents (ThermoFisher Scientific) were used to determine Total ROS and superoxide production as described before [43]. Data shown are mean ± SEM of three independent experiments.

### Statistical analysis

All data were representative of at least three technical repeats. Correlation analysis was performed by using GraphPad 5.0 software and the non-parametric Spearman *r* correlation test with a two-sided *t*-test for significance. The rest of the data were shown as mean ± SEM and the mean values were compared using Student’s *t*-tail test. Values of *P* < 0.05 and *P* < 0.01 were considered statistically significant.

## Supplementary information

Supplementary Figure Legends

Supplementary Figure 1

Supplementary Figure 2

Supplementary Figure 3

Supplementary Figure 4

Supplementary Figure 5

Supplementary Figure 6

Supplementary Figure 7

Supplementary Figure 8

Supplementary Figure 9

Supplementary Figure 10

Supplementary Figure 11
