# Supplementary material for: Mitochondrial estrogen receptors alter mitochondrial priming and response to endocrine therapy in breast cancer cells
Source: Cell Death Discov. 2021 Jul 22;7:189. doi: 10.1038/s41420-021-00573-2 (PMC8298581; doi:10.1038/s41420-021-00573-2)
Supplement: Supplementary file 1 — Supplementary Figure Legends [file 41420_2021_573_MOESM1_ESM.docx]

**Supplementary Figure S1.** ER-α and ER-β expression profiles in ATCC breast cancer cell line panel. A) Protein expression status of ER-α/-β in breast cancer cells and normal breast epithelial cells were determined by using immunoblotting. Two different antibodies were used for analyzing ER-α expression in breast cancer cells (ER-α (#8644, Cell Signaling) and ER-α (#sc-8002, Santa Cruz)). B) mRNA levels of ER-α/-β in breast cancer cells and normal breast epithelial cells were determined by using one-step RT-qPCR. Data were shown as heat-map graphs.

**Supplementary Figure S2.** Normal breast epithelial cells were comparatively less primed than breast cancer cell lines. A) MCF10F, MCF10A, MCF12A and 184B5 cells were treated with indicated concentrations of tamoxifen, fulvestrant and anastrozole for 48 hours and cell viability was evaluated by CellTiter Glo-assay (mean±SEM, n=3). EC_50_ values were determined by nonlinear regression analysis and pEC_50_ values were shown. B) Heat-map of BH3 profiles of normal breast epithelial cells (peptide concentration: 10 μM, n=3). C) Heat-map of dBH3 profiles of normal breast epithelial cells following treatment with EC_50_ values of tamoxifen, anastrozole and fulvestrant for 16 hours (peptide concentration: 1 μM BIM, n=3).

**Supplementary Figure S3.** Endocrine therapies do not induce apoptosis at EC_50_ concentrations in ER-α-positive and ER-α-negative breast cancer cells. A) MCF7, HCC1500, ZR-75-1, CAMA-1, UACC-893, HCC1954, BT-20 and MDA-MB-436 cells were treated with EC_50_ concentrations of tamoxifen, fulvestrant and anastrozole for 0-72 hours. Proliferation of cells was monitored by CyQuant NF proliferation assay (mean±SEM, n=4). B) MCF7, HCC1500, ZR-75-1, CAMA-1, UACC-893, HCC1954, BT-20 and MDA-MB-436 cells were treated with EC_50_ concentrations of tamoxifen, fulvestrant and anastrozole for 16 hours. Cell cycle profiles of untreated and treated cells were determined by BD Cycletest Plus DNA assay kit on FACSCanto flow cytometer. Data were shown as % of cells in G2/M, S and G1 phases representing three independent experiments. C) MCF7, HCC1500, ZR-75-1, CAMA-1, UACC-893, HCC1954, BT-20 and MDA-MB-436 cells were treated with EC_50_ concentrations of tamoxifen, fulvestrant and anastrozole for 48 hours. Staurosporine (0.5 μM) was used as positive control to induce apoptotic cell death in breast cancer cells. Apoptosis was analyzed by using Annexin V/PI staining.

**Supplementary Figure S4.** BH3 profiles of breast cancer cells with 100 μM peptide concentration. A) Heat-map of BH3 profiles of breast cancer cells (peptide concentration: 100 μM, n=3). B) Correlation of BH3 profiles (BID, BIM, BAD, PUMA, BMF, NOXA and HRK) of 40 breast cancer cell lines with EC_50_ values of tamoxifen, anastrozole and fulvestrant was determined by non-parametric Spearman r correlation test with a two-sided t-test for significance.

**Supplementary Figure S5.** Acquired resistance to endocrine therapies in breast cancer cells led to diminished anti-proliferative response. A) HCC70, HCC70-TAM_R_, CAMA-1, CAMA-1-TAMR, HCC1395, HCC1395-FULV_R_, MDA-MB-415, MDA-MB-415-FULV_R_, MDA-MB-361, MDA-MB-361-ANA_R_, MDA-MB-415 and MDA-MB-415-ANA_R_ cells were treated with EC_50_ concentrations of tamoxifen, fulvestrant and anastrozole for parental HCC70, CAMA-1, HCC1395, MDA-MB-415 and MDA-MB-361 cells. Following treatment for 0-72 hours, cells proliferation was detected by CyQuant NF proliferation assay (mean±SEM, n=4). B) HCC70, HCC70-TAM_R_, CAMA-1, CAMA-1-TAMR, HCC1395, HCC1395-FULV_R_, MDA-MB-415, MDA-MB-415-FULV_R_, MDA-MB-361, MDA-MB-361-ANA_R_, MDA-MB-415 and MDA-MB-415-ANA_R_ cells were treated with tamoxifen, fulvestrant and anastrozole at EC_50_ concentrations for parental cells (HCC70, CAMA-1, HCC1395, MDA-MB-415 and MDA-MB-361). Following treatment for 48 hours, apoptosis was evaluated by using Annexin V/PI staining (mean±SEM, n=3) C) HCC70, HCC70-TAM_R_, CAMA-1, CAMA-1-TAMR, HCC1395, HCC1395-FULV_R_, MDA-MB-415, MDA-MB-415-FULV_R_, MDA-MB-361, MDA-MB-361-ANA_R_, MDA-MB-415 and MDA-MB-415-ANA_R_ cells were treated with tamoxifen, fulvestrant and anastrozole at EC_50_ concentrations for parental cells (HCC70, CAMA-1, HCC1395, MDA-MB-415 and MDA-MB-361). Following treatment for 16 hours, cell cycle profiles of untreated and treated cells were determined by BD Cycletest Plus DNA assay kit on FACSCanto flow cytometer. Data were shown as % of cells in G2/M, S and G1 phases representing three independent experiments.

**Supplementary Figure S6.** Subcellular localization of ER-α and ER-β in breast cancer cells and normal breast epithelial cells. Cytoplasmic, nuclear and mitochondrial localization of ER-α and ER-β in ZR-75-30, UACC-812, MCF7, MCF10A and MCF-12A was determined by using immunofluorescence staining and confocal microscopy. DAPI and MitoTracker Red CMXRos were used to visualize nucleus and mitochondria of the cells, respectively. Scale bars, 10 μm. Colocalization analysis was performed by using CoLocalizer Pro 3.0.2 software in order to quantify the percent of colocalization of MitoTracker Red CMXRos (pseudocolor red) and Alexa Fluor 488 (pseudocolor green). A sample of colocalization analysis with Manders overlap coefficient (MOC) was show for each cell line. Scatter plots were shown on the upper left corner of the CoLocalizer Pro analysis images. Cytosolic, nuclear and mitochondrial fractions were immunoblotted for ER-α and ER-β. GAPDH, LSD1 and COXIV was probed as loading control for cytosolic, nuclear and mitochondrial fractions, respectively.

**Supplementary Figure S7.** The relationship between mitochondrial localization of ER-α and ER-β with BH3 and dBH3 profiles of breast cancer cells. Correlation of MOC mitochondria/ER-α (A) and MOC mitochondria/ER-β (B) values of 10 ER-α-positive breast cancer cell lines (BT-474, HCC1419, HCC1428, HCC1806, MCF7, MDA-MB-361, T47D, UACC-812, ZR-75-1 and ZR-75-30) with BH3 profiles (BID, BIM, BAD, PUMA, BMF, NOXA and HRK) was determined by non-parametric Spearman r correlation test. C) Correlation of MOC mitochondria/ER-β values of 20 breast cancer cell lines with different ER-α status (BT-474, HCC1419, HCC1428, HCC1806, MCF7, MDA-MB-361, T47D, UACC-812, ZR-75-1 and ZR-75-30, HCC1599, BT-20, MDA-MB-436, HCC70, AU-565, MDA-MB-157, MDA-MB-231, DU4475, HCC1187 and HCC1569) with BH3 profiles (BID, BIM, BAD, PUMA, BMF, NOXA and HRK) was determined by non-parametric Spearman r correlation test. D) Correlation of MOC mitochondria/ER-β values of 10 ER-α-negative breast cancer cell lines (HCC1599, BT-20, MDA-MB-436, HCC70, AU-565, MDA-MB-157, MDA-MB-231, DU4475, HCC1187 and HCC1569) with BH3 profiles (BID, BIM, BAD, PUMA, BMF, NOXA and HRK) was determined by non-parametric Spearman r correlation test (*P<0.05, by two-tailed t test). Correlation of MOC mitochondria/ER-α (E) and MOC mitochondria/ER-β (F) values of 10 ER-α-positive breast cancer cell lines (BT-474, HCC1419, HCC1428, HCC1806, MCF7, MDA-MB-361, T47D, UACC-812, ZR-75-1 and ZR-75-30) with dBH3 profiles was determined by non-parametric Spearman r correlation test (*P<0.05, by two-tailed t test). G) Correlation of MOC mitochondria/ER-β values of 20 breast cancer cell lines with different ER-α status (BT-474, HCC1419, HCC1428, HCC1806, MCF7, MDA-MB-361, T47D, UACC-812, ZR-75-1 and ZR-75-30, HCC1599, BT-20, MDA-MB-436, HCC70, AU-565, MDA-MB-157, MDA-MB-231, DU4475, HCC1187 and HCC1569) with dBH3 profiles was determined by non-parametric Spearman r correlation test (*P<0.05, by two-tailed t test). H) Correlation of MOC mitochondria/ER-β values of 10 ER-α-negative breast cancer cell lines (HCC1599, BT-20, MDA-MB-436, HCC70, AU-565, MDA-MB-157, MDA-MB-231, DU4475, HCC1187 and HCC1569) with dBH3 profiles was determined by non-parametric Spearman r correlation test (*P<0.05, **P<0.01 by two-tailed t test)

**Supplementary Figure S8.** Alteration of cellular response of breast cancer cells to endocrine treatment by mitochondrial estrogen receptors. ER-β-silenced (A) BT-20, (B) MDA-MB-436 (C) HCC1187 and (D) HCC1569 cells were transfected with pCMV-Myc-Mito, pCMV-Myc-ER-β-Mito and pCMV-Myc-ER-α-Mito vectors. Untransfected, scrambled shRNA-transfected, ER-β shRNA-transfected, ER-β shRNA plus pCMV-Myc-Mito-transfected, ER-β shRNA plus pCMV-Myc-ER-β-Mito-transfected and ER-β shRNA plus pCMV-Myc-ER-α-Mito-transfected cells were treated with tamoxifen, fulvestrant and anastrozole at EC_50_ concentrations for parental cells (BT-20, MDA-MB-436, HCC1187 and HCC1569) for 0-72 hours. Cell proliferation was evaluated by CyQuant NF proliferation assay (mean±SEM, n=4).

ER-β-silenced (E) BT-20, (F) MDA-MB-436 (G) HCC1187 and (H) HCC1569 cells were transfected with pCMV-Myc-Mito, pCMV-Myc-ER-β-Mito and pCMV-Myc-ER-α-Mito vectors. Untransfected, scrambled shRNA-transfected, ER-β shRNA-transfected, ER-β shRNA plus pCMV-Myc-Mito-transfected, ER-β shRNA plus pCMV-Myc-ER-β-Mito-transfected and ER-β shRNA plus pCMV-Myc-ER-α-Mito-transfected cells were treated with tamoxifen, fulvestrant and anastrozole at EC_50_ concentrations for parental cells (BT-20, MDA-MB-436, HCC1187 and HCC1569) for 16 hours. cell cycle profiles of untreated and treated cells were determined by BD Cycletest Plus DNA assay kit on FACSCanto flow cytometer. Data were shown as % of cells in G2/M, S and G1 phases representing three independent experiments. ER-β-silenced (I) BT-20, (J) MDA-MB-436 (K) HCC1187 and (L) HCC1569 cells were transfected with pCMV-Myc-Mito, pCMV-Myc-ER-β-Mito and pCMV-Myc-ER-α-Mito vectors. Untransfected, scrambled shRNA-transfected, ER-β shRNA-transfected, ER-β shRNA plus pCMV-Myc-Mito-transfected, ER-β shRNA plus pCMV-Myc-ER-β-Mito-transfected and ER-β shRNA plus pCMV-Myc-ER-α-Mito-transfected cells were treated with tamoxifen, fulvestrant and anastrozole at EC_50_ concentrations for parental cells (BT-20, MDA-MB-436, HCC1187 and HCC1569) for 48 hours. Staurosporine (0.5 μM) was used as positive control for apoptotic cell death. Apoptosis was evaluated by using Annexin V/PI staining.

**Supplementary Figure S9.** Mitochondrial estrogen receptors modulate endocrine therapy response in breast cancer cells. A) ER-α-negative HCC1187, HCC1569, MDA-MB-436 and BT-20 cells were transfected with ER-β shRNA or scrambled shRNA to knockdown ER-β. HCC1187, HCC1569, MDA-MB-436 and BT-20 cells were transfected with pAcGFP1-Mito, pACGFP1-ER-β-Mito and pAcGFP1-ER-α-Mito vectors. Hoechst 33342 and MitoTracker Red CMXRos were used to stain nucleus and mitochondria of the cells, respectively. The expression of pAcGFP1-Mito, pACGFP1-ER-β-Mito and pAcGFP1-ER-α-Mito vectors in HCC1187, HCC1569, MDA-MB-436 and BT-20 cells was evaluated by using EVOS FLoid digital microscopy system. B) Untransfected, scrambled shRNA-transfected, ER-β shRNA-transfected, ER-β shRNA plus pAcGFP1-Mito-transfected, ER-β shRNA plus pAcGFP1-ER-β-Mito-transfected and ER-β shRNA plus pAcGFP1-ER-α-Mito-transfected cells were treated with tamoxifen, fulvestrant and anastrozole for 48 hours and cell viability was evaluated by CellTiter-Glo assay (mean±SEM, n=3). EC_50_ values of cells were determined by nonlinear regression analysis and pEC_50_ values were shown. Heat-map graphs of C) BH3 profiles and D) dBH3 profiles of untransfected, scrambled shRNA-transfected, ER-β shRNA-transfected, ER-β shRNA plus pAcGFP1-Mito-transfected, ER-β shRNA plus pACGFP1-ER-β-Mito-transfected and ER-β shRNA plus pAcGFP1-ER-α-Mito-transfected cells.

**Supplementary Figure S10.** Mitochondrial ER-α and ER-β do not trigger transcriptional activity in breast cancer cells. A) Luciferase reporter assays of ER-α and ER-β transcriptional activity in MCF7 cells transiently transfected with 3X-ERE-TATA-luc. Cells were treated with 10 nM E2, 1 nM PPT, or 1 nM DPN for 24 hours. Data shown are mean ± SEM of 3 independent experiments. B) ER-β-silenced BT-20, MDA-MB-436, HCC1187 and HCC1569 cells were transfected with pCMV-Myc-Mito, pCMV-Myc-ER-β-Mito and pCMV-Myc-ER-α-Mito vectors. Cells were transiently transfected with 3X-ERE-TATA-luc and treated with 10 nM E2, 1 nM PPT, or 1 nM DPN for 24 hours. Data shown are mean ± SEM of 3 independent experiments.

**Supplementary Figure S11.** Mitochondrial ER-β prominently modulates mitochondrial bioenergetics in breast cancer cells. OCR values of non-mitochondrial oxygen consumption, basal respiration, maximum respiration, proton leak, ATP production and spare respiratory capacity were calculated in untransfected, scrambled shRNA-transfected, ER-β shRNA-transfected, ER-β shRNA plus pCMV-Myc-Mito-transfected, ER-β shRNA plus pCMV-Myc-ER-β-Mito-transfected and ER-β shRNA plus pCMV-Myc-ER-α-Mito-transfected A) HCC1187, B) HCC1569, C) MDA-MB-436 and D) BT-20 cells (mean±SEM, n=3; *P<0.05, **P<0.01 by two-tailed Student’s t test, Untransfected vs. ER-β shRNA, ER-β shRNA vs. ER-β shRNA/pCMV-Myc-ER-β-Mito).
