# Supplementary figures and images for: Mitochondrial estrogen receptors alter mitochondrial priming and response to endocrine therapy in breast cancer cells
Source: Cell Death Discov. 2021 Jul 22;7:189. doi: 10.1038/s41420-021-00573-2 (PMC8298581; doi:10.1038/s41420-021-00573-2)

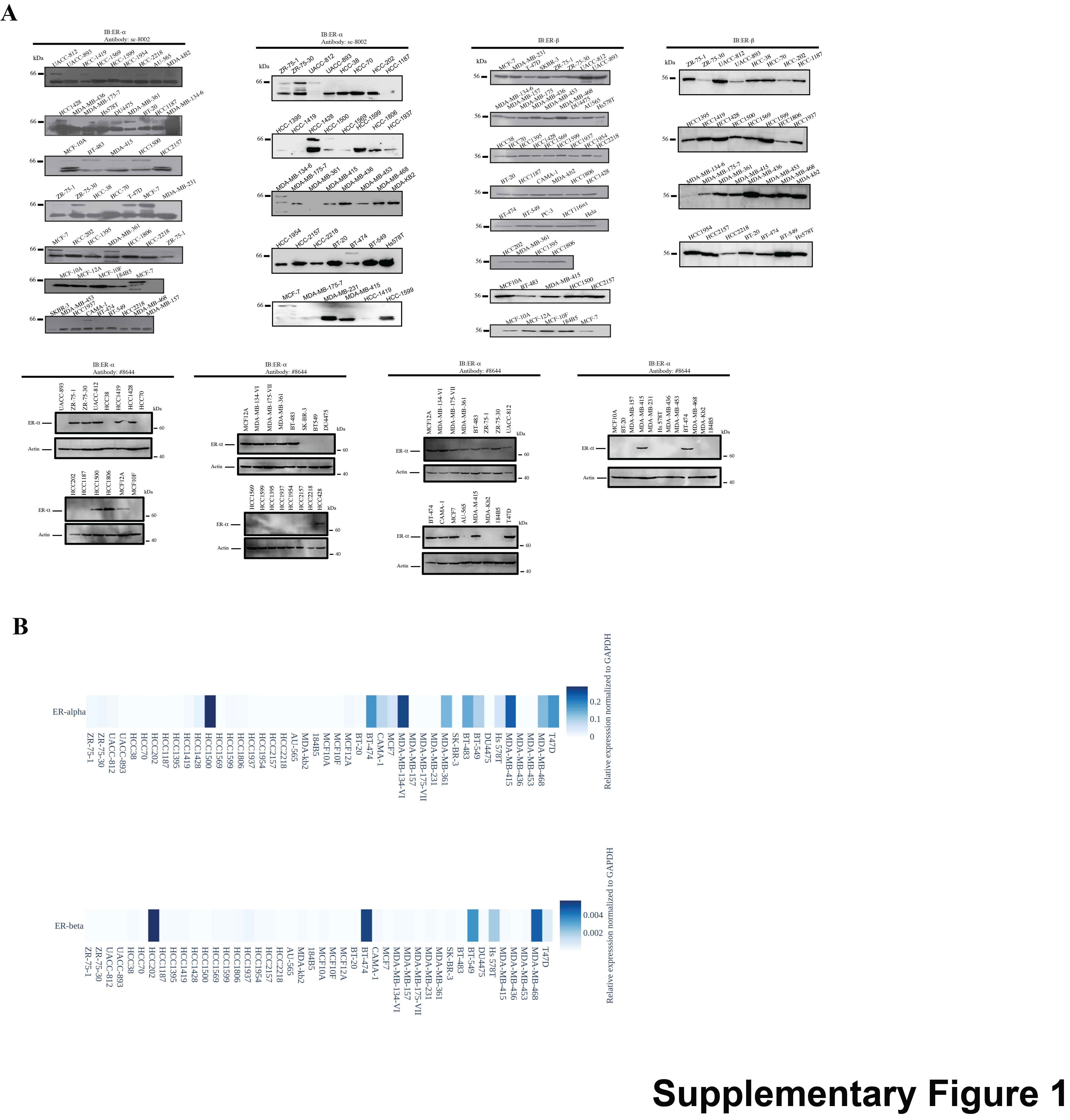

Supplement: Supplementary file 2 — Supplementary Figure 1 [file 41420_2021_573_MOESM2_ESM.png]

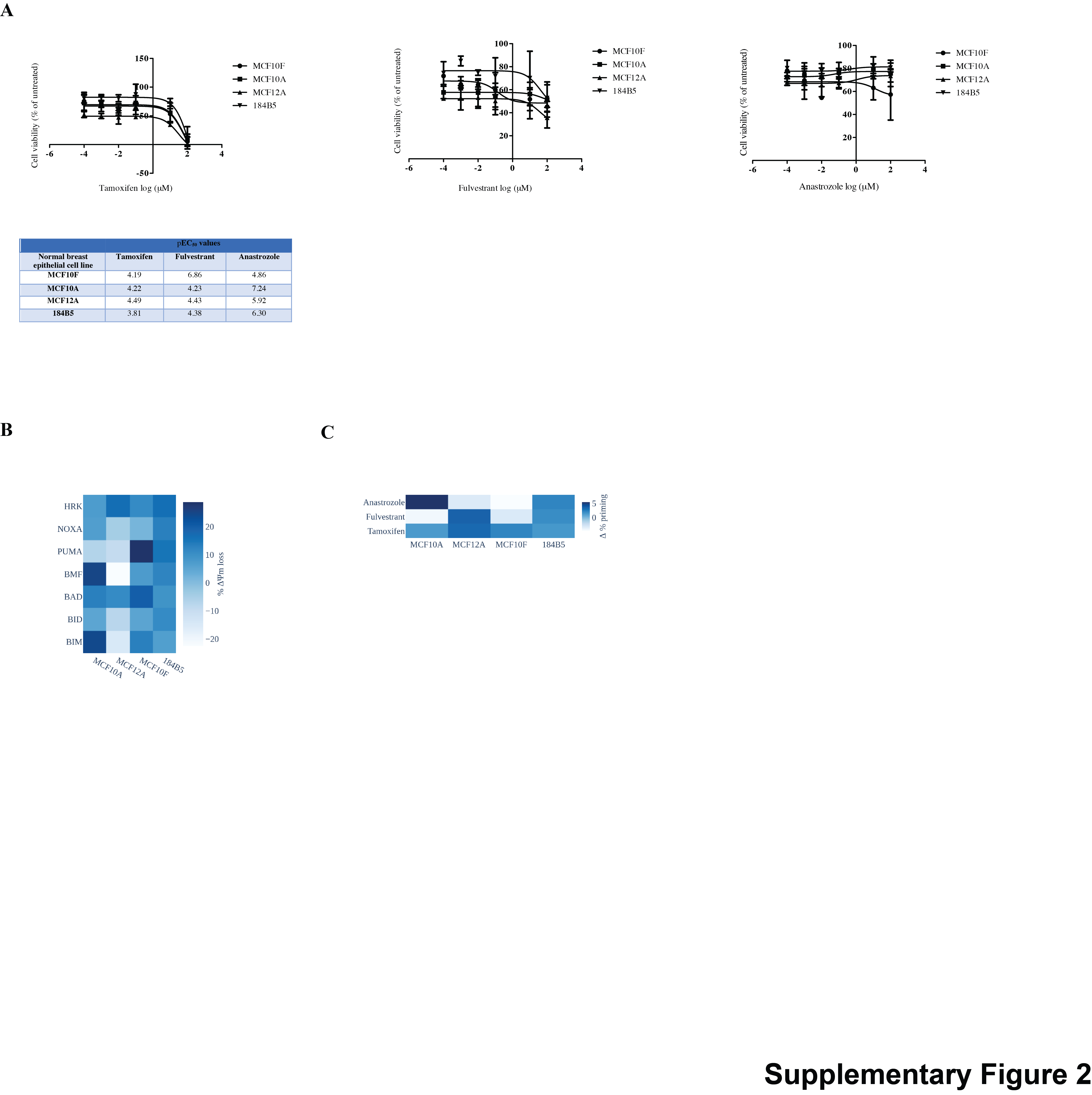

Supplement: Supplementary file 3 — Supplementary Figure 2 [file 41420_2021_573_MOESM3_ESM.png]

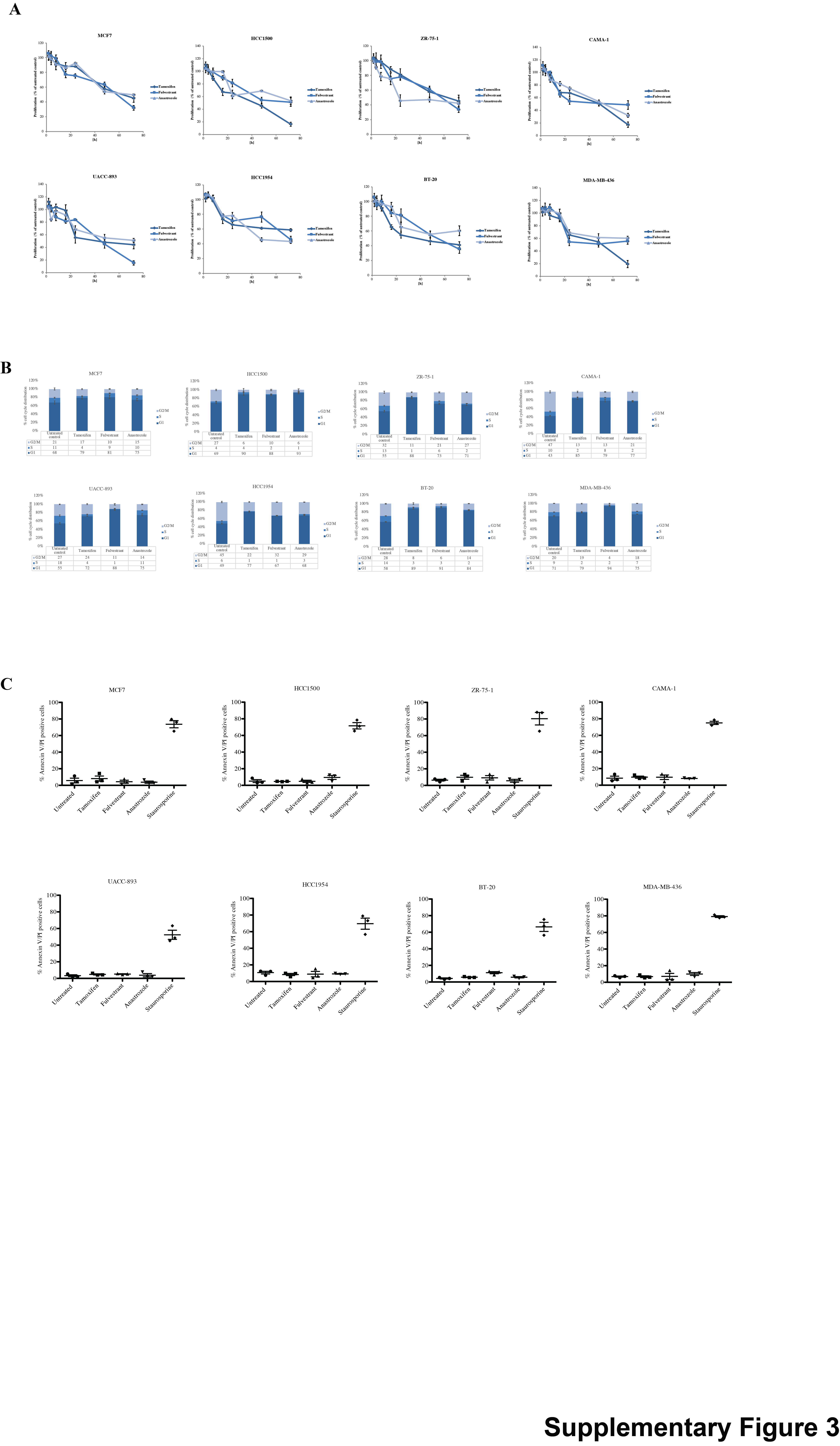

Supplement: Supplementary file 4 — Supplementary Figure 3 [file 41420_2021_573_MOESM4_ESM.png]

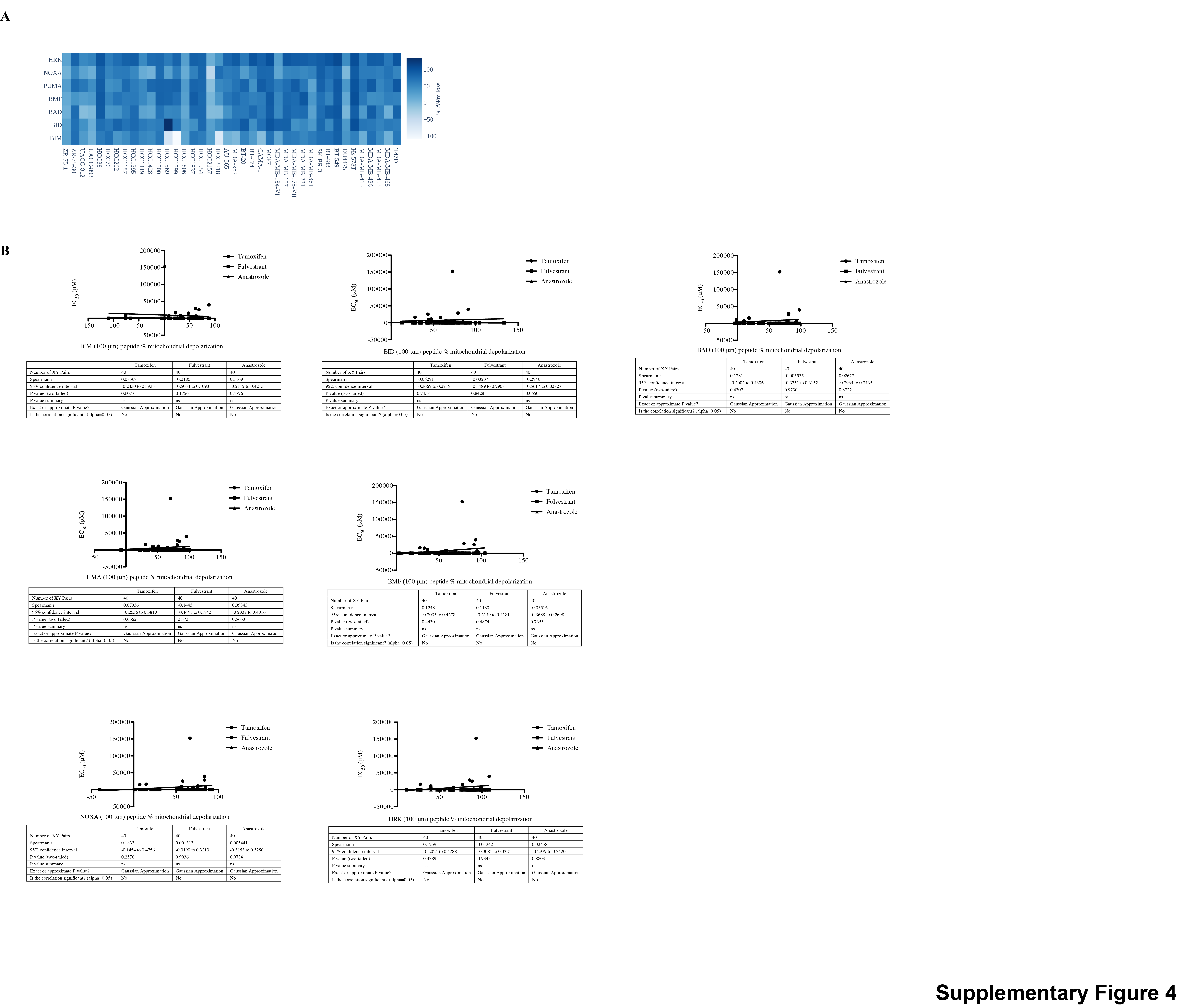

Supplement: Supplementary file 5 — Supplementary Figure 4 [file 41420_2021_573_MOESM5_ESM.png]

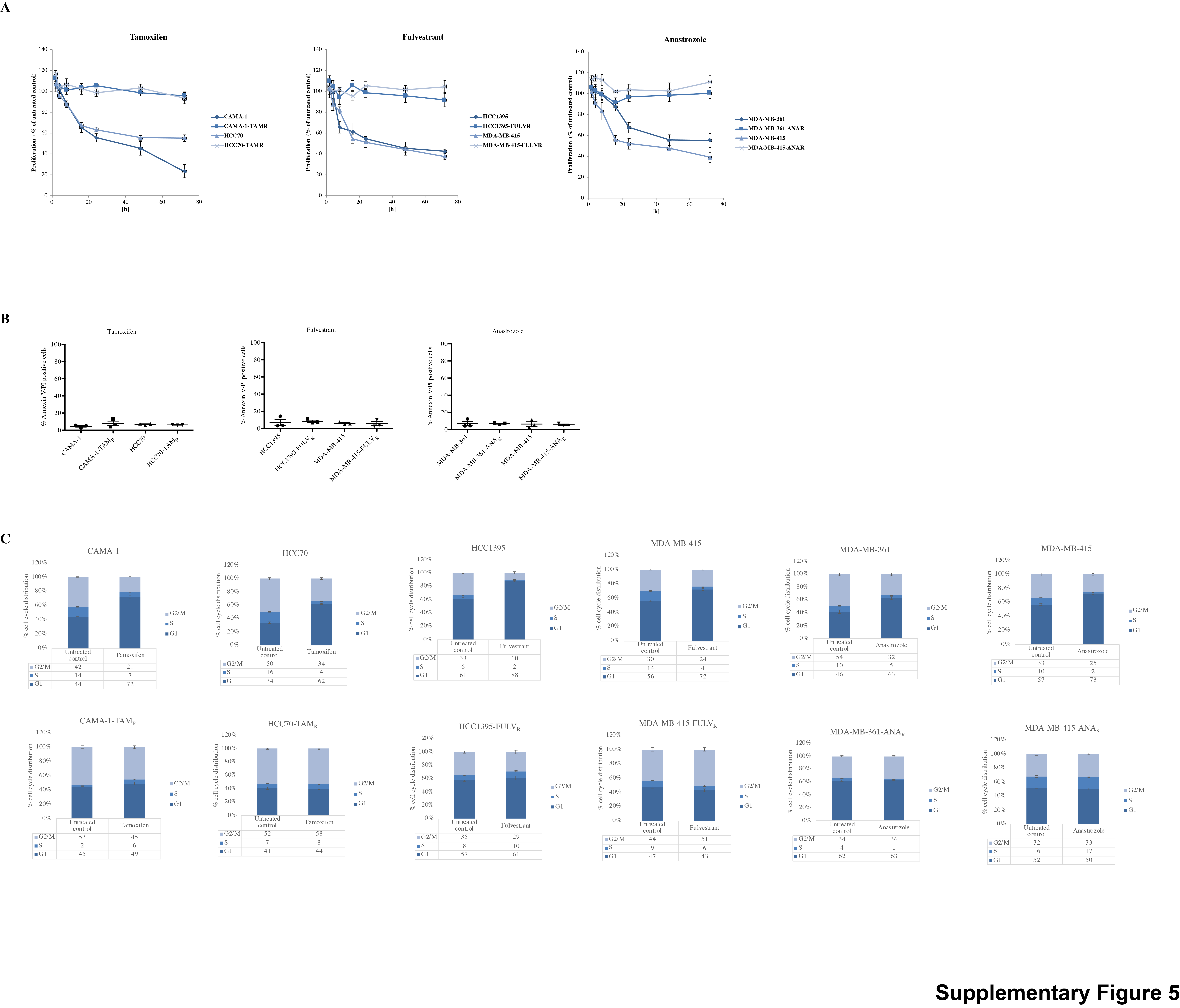

Supplement: Supplementary file 6 — Supplementary Figure 5 [file 41420_2021_573_MOESM6_ESM.png]

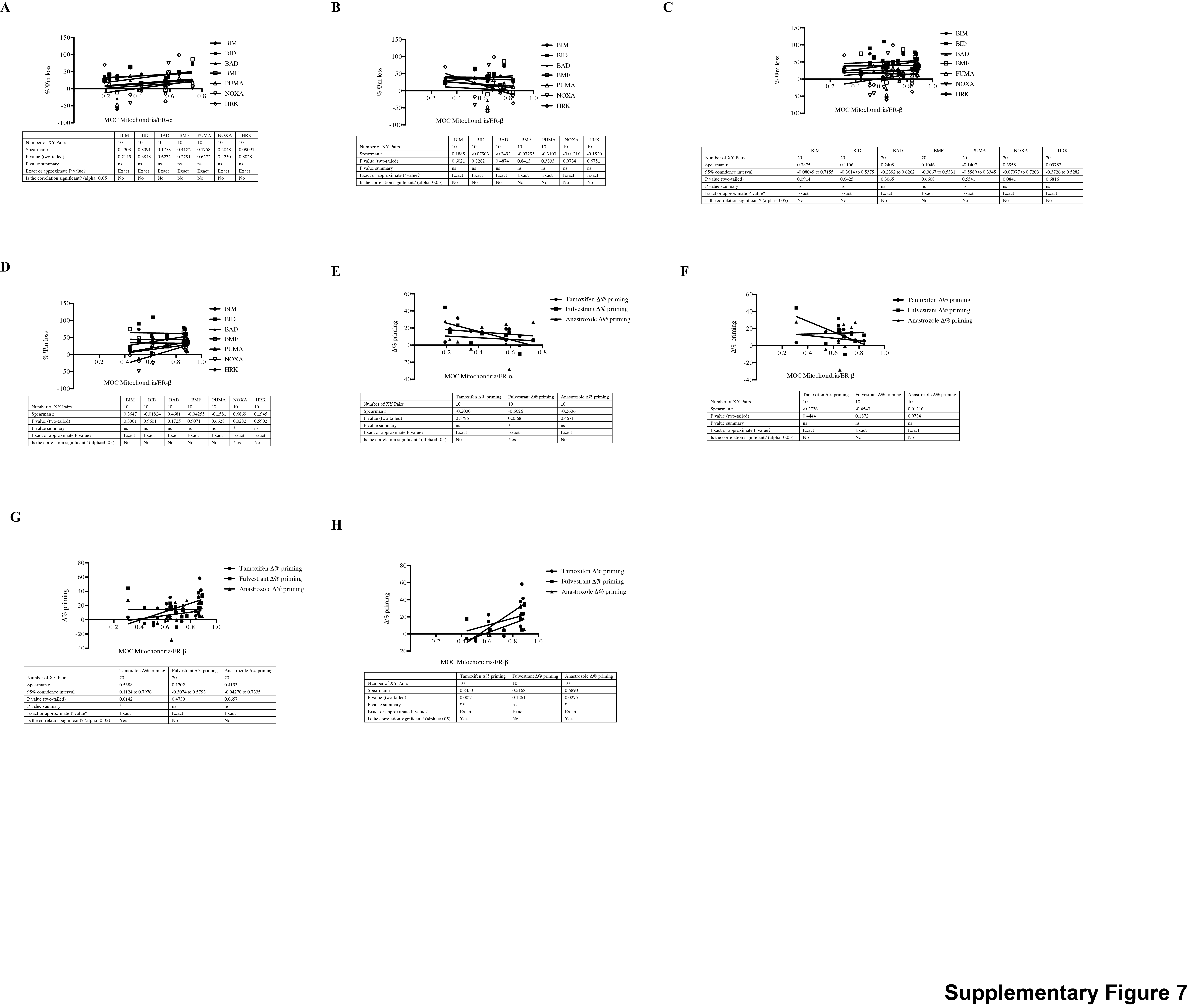

Supplement: Supplementary file 8 — Supplementary Figure 7 [file 41420_2021_573_MOESM8_ESM.png]

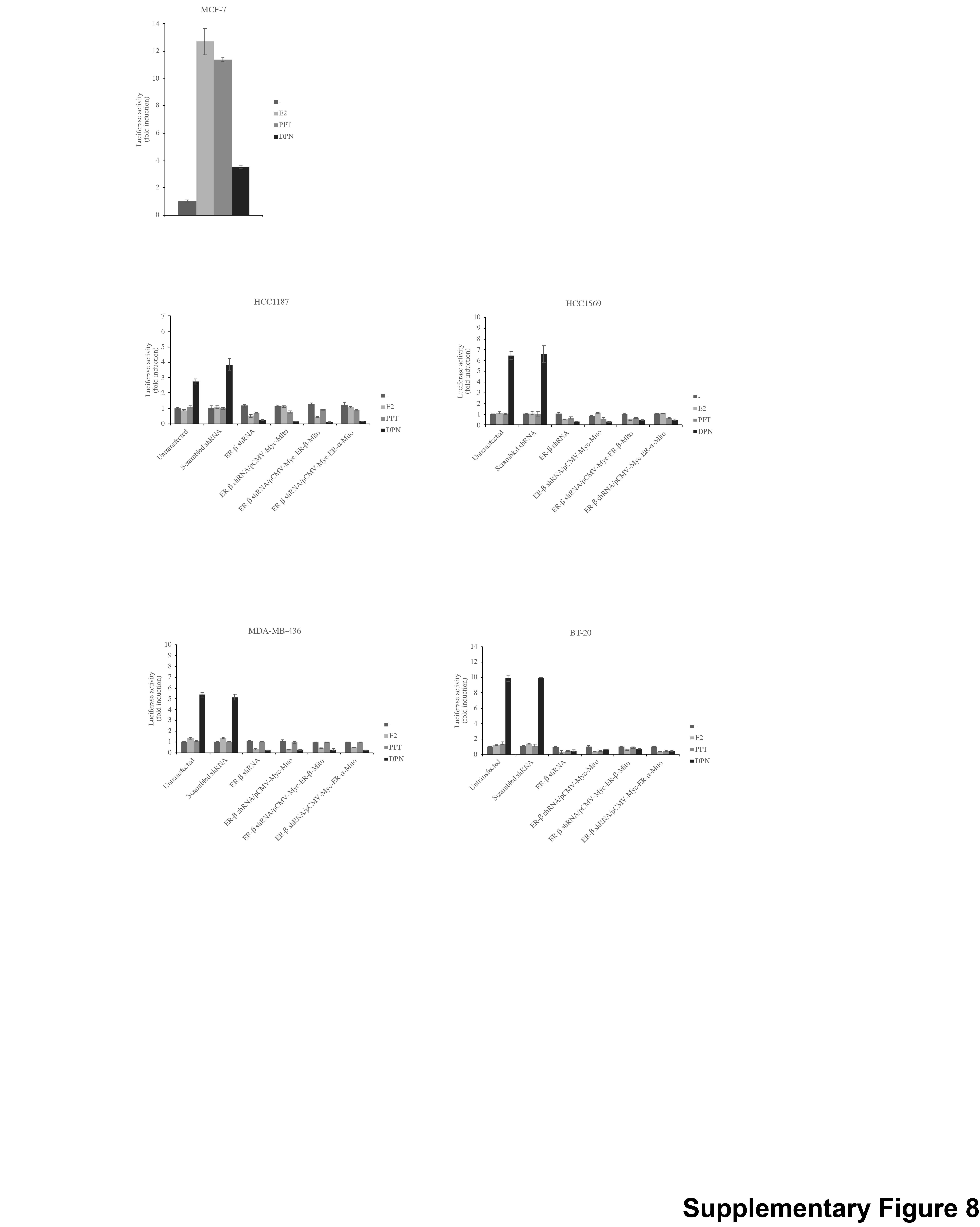

Supplement: Supplementary file 9 — Supplementary Figure 8 [file 41420_2021_573_MOESM9_ESM.png]
